# A genetic relationship between nitrogen use efficiency and seedling root traits in maize as revealed by QTL analysis

**DOI:** 10.1093/jxb/erv127

**Published:** 2015-04-06

**Authors:** Pengcheng Li, Fanjun Chen, Hongguang Cai, Jianchao Liu, Qingchun Pan, Zhigang Liu, Riliang Gu, Guohua Mi, Fusuo Zhang, Lixing Yuan

**Affiliations:** ^1^Key Laboratory of Plant-Soil Interaction, MOE, Center for Resources, Environment and Food Security, College Resources and Environmental Sciences, China Agricultural University, Beijing, China 100193

**Keywords:** Genetic relationship, nitrogen uptake efficiency, nitrogen use efficiency, QTL analysis, QTL clusters, roots, root system architecture, *Zea mays* L.

## Abstract

That root system architecture (RSA) has an essential role in nitrogen acquisition is expected in maize, but the genetic relationship between RSA and nitrogen use efficiency (NUE) traits remains to be elucidated. Here, the genetic basis of RSA and NUE traits was investigated in maize using a recombination inbred line population that was derived from two lines contrasted for both traits. Under high-nitrogen and low-nitrogen conditions, 10 NUE- and 9 RSA-related traits were evaluated in four field environments and three hydroponic experiments, respectively. In contrast to nitrogen utilization efficiency (NutE), nitrogen uptake efficiency (NupE) had significant phenotypic correlations with RSA, particularly the traits of seminal roots (r = 0.15–0.31) and crown roots (r = 0.15–0.18). A total of 331 quantitative trait loci (QTLs) were detected, including 184 and 147 QTLs for NUE- and RSA-related traits, respectively. These QTLs were assigned into 64 distinct QTL clusters, and ~70% of QTLs for nitrogen-efficiency (NUE, NupE, and NutE) coincided in clusters with those for RSA. Five important QTLs clusters at the chromosomal regions bin1.04, 2.04, 3.04, 3.05/3.06, and 6.07/6.08 were found in which QTLs for both traits had favourable effects from alleles coming from the large-rooted and high-NupE parent. Introgression of these QTL clusters in the advanced backcross-derived lines conferred mean increases in grain yield of ~14.8% for the line *per se* and ~15.9% in the testcross. These results reveal a significant genetic relationship between RSA and NUE traits, and uncover the most promising genomic regions for marker-assisted selection of RSA to improve NUE in maize.

## Highlight

This research determined the significant genetic and phenotypic relationships between seedling root traits and nitrogen use efficiency (NUE), and further identified five QTL clusters for improving NUE in maize.

## Introduction

Nitrogen (N) is quantitatively the most important mineral nutrient for plant growth and development. In the past decades application of synthetic N fertilizer has increased crop yield significantly. However, N fertilizer production consumes ~1% of the world’s total annual energy supply and results in significant amounts of greenhouse gas emissions (Smith, 2002; Zhang *et al.*, 2013). Moreover, the overuse of N fertilizer in many regions of the world causes serious damage to the environment, including soil acidification, and water and air pollution (Galloway *et al.*, 2008; Guo *et al.*, 2010; Liu *et al.*, 2013). To simultaneously ensure food security and environmental quality, it is important to cultivate crops that are able to uptake and utilize N efficiently (Good and Beatty, 2011).

Maize (*Zea mays* L.) is one of the world’s major crops and ~967 million metric tons were produced in 2013 for food, feed, and industrial uses (Ort and Long, 2014). Meanwhile, global maize production consumes almost one-fifth of total N fertilizer (FAO, 2000). In terms of N use efficiency (NUE), genetic variation in the maize germplasm implies that the selection of better NUE varieties can be achieved by breeding processes (Paponov *et al.*, 2005; Uribelarrea *et al.*, 2007). However, developing maize cultivars for NUE traits is challenging because of the genetic complexity and strong interaction with the environment. The general definition of NUE is plant yield in grain per unit of available N in soils (Moll *et al.*, 1982). NUE consists of two main components: N uptake efficiency (NupE), the ability of plants to remove N from the soil; and N utilization efficiency (NutE), the ability of plants to use N to produce grain yield (GY). Correlation studies in maize between these components of NUE have revealed that variation in NupE likely contributes more to variation in NUE under both high-N and low-N conditions, while NutE contributes more at the low-N input (Mi *et al.*, 1998; Bertin and Gallais, 2000; Presterl *et al.*, 2002). In addition, in regions where N fertilizer is overused, maize cultivars with high NupE can help accumulate excess N and subsequently reduce N leaching into the environment.

Roots are essential for the acquisition of mineral nutrients, including N. Effective root system architecture (RSA) is important in breeding maize genotypes for high NupE and helping prevent N leaching (Mackay and Barber 1986). In maize, the hypothetical ideotype RSA for efficient N acquisition has been proposed in several physiological studies on different genotypes (Mi *et al.*, 2010; Lynch, 2013). In general, increases in root size (root dry weight, root length, and root density) improve N uptake ability and yield formation in maize (Chen *et al.*, 2013; Mu *et al.*, 2015). Given the considerable carbon costs for root growth, the optimal number of crown roots (CRs) and lateral roots (LRs) is essential for N acquisition from low-N soil (Trachsel *et al.*, 2013; Postma *et al.*, 2014; Saengwilai *et al.*, 2014). Besides the morphology, the architecture of roots also plays an important role in N acquisition; for example, a steeper and deeper root more efficiently absorbs N in deep soil layers (Wiesler and Horst 1994; Lynch, 2013; Trachsel *et al.*, 2013). In addition, maize RSA can be strongly influenced by the N availability in the soil to efficiently capture N resources. Under low-N conditions, maize plants reduce the number of CRs but increase the total root length (Feil *et al.*, 1990; Chun *et al.*, 2005; Liu *et al.*, 2008). In N-rich soil, the LRs are stimulated to branch and elongate (Granato and Raper, 1989; Liu *et al.*, 2010). Thus, the components of RSA themselves and their plasticity to soil N availability deserve important consideration as potential traits for genetic improvement of NupE in maize.

Both NUE and RSA are complex traits, depending on both genetic and environmental factors and their interactions. In maize, poor understanding of the genetic basis of NUE as well as limited knowledge about RSA and its relationship with NUE have hindered selection efficiency of RSA-based NUE traits. Recently, mapping quantitative trait loci (QTL) has become a powerful tool to identify genomic regions and even putative candidate genes involved in the genetic variation of complex traits. Many maize QTLs for NUE traits at agronomic and physiological levels have been identified, including traits for NupE and NutE (Bertin and Gallais, 2000; Coque and Gallais 2006; Coque *et al.*, 2008), N grain uptake (Coque *et al.*, 2006), post-silking N-uptake (Gallais and Hirel, 2004; Coque *et al.*, 2008), N remobilization (Gallais and Hirel, 2004; Coque *et al.*, 2006; Coque *et al.*, 2008), and N metabolism (Zhang *et al.*, 2010; Cañas *et al.*, 2012). Likewise, many maize QTLs that regulate RSA have been identified in several linkage populations, and meta-analysis has further determined the putative consensus root-QTL clusters (Lebreton *et al.*, 1995; Landi *et al.*, 2002; Tuberosa *et al.*, 2002, 2003; Zhu *et al.*, 2006; Liu *et al.*, 2008; Hund *et al.*, 2011). Despite these extensive advancements in the genetic knowledge of RSA and NUE, it is not clear if genetic relationships between, and common QTLs for, both traits exist. Guingo *et al.* (1998) evaluated RSA traits for root lodging at flowering stage in the field using a maize recombination inbred line (RIL) population. Although some of the identified QTLs for root density, diameter, and dry weight were later found to co-localize with QTLs for NUE, the correlations between RSA and NUE at the phenotypic level have not been reported (Coque *et al.*, 2008). Additionally, because it easy to select for root traits at the seedling stage in breeding programmes, establishing the relationship between RSA and NUE at the seedling stage is more promising than at others stages. Therefore, a deep investigation is required to directly uncover the genetic relationship between seedling root traits and NUE traits in a population specific to RSA, and also in response to different levels of N supply.

In the present study, a maize RIL population derived from a cross of two parental lines Ye478 and Wu312 with contrasting NUE and RSA traits was evaluated (Liu *et al.*, 2009). Phenotyping of NUE and RSA in response to two N levels was performed for adult plants in field trials and seedling plants in hydroponics, respectively. The study objectives were to (i) investigate the phenotypic association between RSA and NUE traits under high N (HN) and low N (LN) conditions, (ii) perform QTL mapping and further determine the QTL clusters for RSA and NUE traits, and (iii) validate the breeding value of RSA-QTLs for improving NUE traits at *per se* and hybrid levels in maize.

## Materials and methods

### Plant materials

The RIL population, consisting of 218 F8 lines, was derived from a cross between two inbred lines, Ye478 (female parent) and Wu312 (male parent), as described by Liu *et al.* (2011). An advanced-backcross line (ABL) population, consisting of 187 BC_4_F_3_ lines, was generated from advanced backcross processes using Ye478 as the donor parent and Wu312 as the recurrent parent (Liu *et al.*, 2011; Cai *et al.*, 2012). Each BC_4_F_3_ line was further crossed with a tester line 178 to produce the F1 testcross population. Ye478 was developed in China during the 1990s and was the female parent of more than 50 high-yielding hybrids (Yu and Zhu 1996; Li *et al.*, 2005). Wu312 was derived from an unknown hybrid. In previous studies, Ye478 was characterized as a nitrogen use efficient inbred, and Wu312 as a nitrogen use inefficient inbred (Tian *et al.*, 2006; Liu *et al.*, 2009; Peng *et al.*, 2010). Ye478 had higher GYs and shoot biomass, and took up more N in both HN and LN conditions than Wu312. The root system of Ye478, as indicated by root length, root number, root biomass, and root to shoot ratio, was larger than Wu312 both in hydroponics and field conditions.

### Field experiments

Field experiments for 218 RILs and the two parent lines were conducted at six environments, including the China Agricultural University (CAU, Beijing, China) experimental station in Dongbeiwang (DBW) (40°03′N, 116°18′E, 60 m above sea level) in 2006 and 2007, at the CAU experimental station in Changping (CP) (40°06′N, 116°19′E, 45 m above sea level) in 2007 and 2008, and at the CAU experimental station in Shangzhuang (SZ) (40°06′N, 116°11′E, 46 m above sea level) in 2009 and 2010 (Supplementary Table S1). In SZ, field experiments for an advanced 187 BC_4_F_3_ line population and the two parents, together with the testcross F1 hybrids, were also performed for two years (2009 and 2010). The nutritional composition of the soils before sowing in each field and the information of fertilizer treatments are summarized in Supplementary Table S1. Before sowing, the fields were supplied with 135kg/ha KCl, and with 750kg/ha CaH_4_O_8_P_2_ in DBW and SZ, and 422kg/ha in CP. The N supply in soils varied from 37–84kg/ha across the six environments, representing a mild LN condition. For the HN treatment, an additional urea-based N fertilizer (391kg/ha for DBW and SZ, and 290kg/ha for CP) was applied (40% of N applied before sowing and 60% at the V9 stage). In addition to soil residual and fertilizer N, other N supplies from soil mineralization and atmosphere deposition could have been available under both HN and LN conditions, but were not taken into account here.

At each location the population was evaluated in a completely randomized block design of one-row plots with three replicates. In DBW and SZ, each row was 4 m long, 0.5 m wide, and contained 13 plants. The planting density was 60,000 plants per hectare. In CP, each row was 2 m long, 0.5 m wide, and contained 11 plants. The planting density was 100,000 plants per hectare. Standard cultivation management practices were performed. The anthesis date (AD) of the two parental lines was similar, whereas those within the RIL population varied by 3 weeks. At maturation stage, the healthy plants within each row (11–13 individuals) were harvested and pooled for the measurements as described in Table 1. Ears were air-dried and then threshed to determine GY. Stover tissues were over-dried for determining stover yield (SY). Nitrogen concentration in grain (GNC) and stover (SNC) was measured using a standard Kjeldahl procedure. NUE, NupE, NutE, harvest index (HI), and nitrogen harvest index (NHI) were calculated as described in Table 1. GY and NUE were evaluated across six environments (E1–E6), whereas other traits were evaluated across four environments (E1–E4). Considering the N supply was constant, the complete correlation between GY and NUE, and between nitrogen uptake (Nup) and NupE, was expected. Therefore, GY and NUE were integrated into a single trait (GY/NUE), and Nup and NupE into a single trait (Nup/NupE), in the phenotypic correlation and principal component analysis (PCA). Because the same QTLs for GY and NUE, and for Nup and NupE, were also expected, GY and Nup traits were omitted from the subsequent QTL analysis.

**Table 1.** Summary of the investigated traits in this study and the measurements

Classification	Trait	Abbreviations	Units	Trait measurements
NUE-related traits	Grain yield	GY	g m^-2^	—
	Nitrogen use efficiency	NUE	g/g	Grain yield/total amount of nitrogen supply
	Nitrogen uptake	Nup	g m^-2^	GY × GNC + SY × SNC
	Nitrogen uptake efficiency	NupE	g/g	Total N uptake/total amount of nitrogen supply
	Nitrogen utilization efficiency	NutE	g/g	Grain yield/total nitrogen content
	Stover yield	SY	g m^-2^	—
	Harvest index	HI	g/g	GY/(GY + SY)
	Grain nitrogen concentration	GNC	%	Kjeldahl method
	Stover nitrogen concentration	SNC	%	Kjeldahl method
	Nitrogen harvest index	NHI	g m^-2^/ g m^-2^	GY × GNC /(GY × GNC + SY × SNC)
RSA-related traits	The number of seminal roots	SRN	number	Average number of the two plants
	The number of crown roots	CRN	number	Average number of the two plants
	The number of lateral roots	LRN	number	Count number of the lateral root within 5cm from the first emerged lateral root in the primary root.
	The length of primary roots	PRL	cm	Measured with a ruler
	The length of seminal roots	SRL	cm	Measured with a ruler
	The length of crown roots	CRL	cm	Measured with a ruler
	Root dry weight	RDW	mg plant^-1^	Dried and weighted using a balance (1/1000g)
	Shoot dry weight	SDW	mg plant^-1^	Dried and weighted using a balance (1/1000g)
	Root to shoot ratio	R/S	mg/mg	Root dry weight/shoot dry weight

### Hydroponics experiments

Maize seeds were sterilized for 20min in a 10% solution of H_2_O_2_, washed with distilled water, soaked in saturated CaSO_4_ for 6h, and then germinated in the dark on moist filter paper at room temperature. After 2 days the germinated seeds were wrapped in a moist filter paper roll and grown until the stage of two visible leaves. The uniform seedlings were then selected and transferred into a plastic tub (60×40×15cm, length × width × height) containing 40L nutrient solution. The distance between two neighbouring plants was 3.3cm in the row and 2.8cm in the column. For HN treatment, the nutrient solution consisted of (mM): 2.0mM Ca(NO_3_)_2_, 0.75 K_2_SO_4_, 0.65 MgSO_4_, 0.1 KCl, 0.25 KH_2_PO_4_, 1×10^−3^ H_3_BO_3_, 1×10^−3^ MnSO_4_, 1×10^−4^ CuSO_4_, 1×10^−3^ ZnSO_4_, 5×10^−6^ (NH_4_)_6_Mo_7_O_24_, 0.1 Fe-EDTA, with pH 6.0. For LN treatment, the 2.0mM Ca(NO_3_)_2_ was replaced by 0.02mM Ca(NO_3_)_2_ plus 1.98mM CaCl_2_ in the first week, and was then substituted by 2.0mM CaCl_2_ until the harvest. The nutrient solution was renewed every 2 days and aerated by a pump. The maize seedlings were grown in a growth chamber with controlled conditions: 28/22°C during a 14/10h light/dark cycle, with a light density of 250–300 μmol m^−2^s^−1^ that was measured at canopy height.

The hydroponically grown two parent lines and 218 RILs were evaluated by three independent experiments (E7–E9). Each experiment was conducted in a completely randomized design with three replicates for two N treatments. The value of each replicate was represented by the means of two seedlings for each RIL or six seedlings for each parental line. The seedlings were harvested at the developmental stage with five to six visible leaves (~20 days after seed germination), and the plants grown under LN condition showed typical N-deficiency symptoms. Roots were separated from the shoot and stored at −20°C before the measurements as described in Table 1. The seminal root numbers (SRN) and crown root numbers (CRN) were counted. The number of lateral roots (LRN) was evaluated within a 5cm region of the primary root starting from the first emerged LR. The primary root length (PRL), seminal root length (SRL), and crown root length (CRL) was measured with a ruler. The roots and shoots were oven-dried at 70°C until a constant weight, and root dry weight (RDW), shoot dry weight (SDW), and root to shoot ratio (R/S) were evaluated.

### Data analysis

Phenotypic data was analysed with software SAS 9.0 (SAS Institute Inc., NC, USA) using the GLM procedure. Combinations of year-location were treated as environments (E). Genotype (G) was treated as ﬁxed, and E and interaction of genotype-by-environment (G × E) as random. The procedure LSMEANS was then performed to estimate phenotype values for the genotypes that were used for the subsequent phenotypic analysis and correlation analysis. The procedure VARCOMP was conducted to estimate genotypic variance $\left(\sigma_{G}^{2}\right)$, G–E interaction variance $\left(\sigma_{G\times E}^{2}\right)$ and error variance $\left(\sigma_{e}^{2}\right)$. The broad-sense heritability (*h* ^2^) of each measured trait was calculated as previously described by Hallauer and Miranda (1981). Pearson correlation coefﬁcients and PCA were calculated using SPSS Statistics 17.0 (SPSS, Inc., Chicago, IL, USA) and further visualized using the R package (R Development Core Team, 2013).

Detection of QTL was performed by the composite interval mapping method (Zeng, 1994) using the software Windows QTL Cartographer version 2.5 (Model 6, Wang *et al.*, 2005). The genotypic data for the RIL population was obtained from Liu *et al.* (2011), showing that a molecular map comprising 184 simple sequence repeat markers covered 2084.1 cM on 10 chromosomes with an average interval of 11.3 cM. Forward regression was analysed using a window size of 10 cM, a walk speed of 2 cM and five control markers. The threshold limit of detection (LOD) values were determined with 1000× permutations at *P* < 0.05 level (Churchill and Doerge, 1994), and LOD thresholds were set at 2.5 for all investigated traits. QTL positions were assigned at the point of maximum LOD score. A QTL meta-analysis was used to investigate coincidences of several QTLs for different traits in a given genetic population, and subsequently, to identify the QTL clusters. Within a cluster, the coincidence of QTLs for two traits was considered to be positive if the allele effect of both QTLs from the same parent showed the same sign, whereas it was considered negative if the two signs were different (Coque *et al.*, 2008). The software MetaQTL Version 1.0 was applied to detect QTL clusters, according to the procedure initiated by Goffinet and Gerber (2000) and further improved by Veyrieras *et al.* (2007).

## Results

### Evaluation of NUE-related traits in the field

**Fig. 1.**
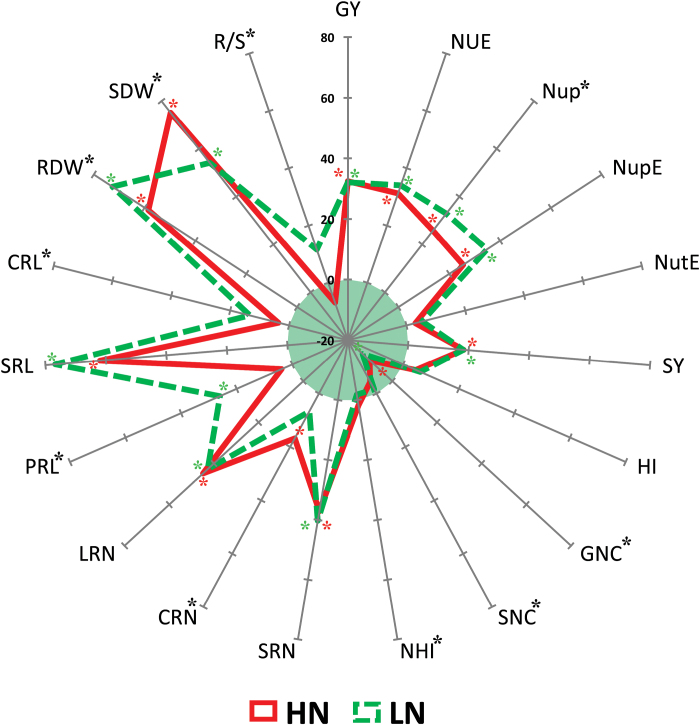
The phenotypic difference between the two parental lines Ye478 and Wu312 under HN and LN levels. The difference for each trait is indicated by the relative increase or decrease of Ye478 to Wu312, which was calculated as (Ye478 − Wu312)/Wu312×100%. The red solid line and green dotted line represent the percentages under HN and LN levels, respectively. A signiﬁcant difference between the two parents at HN level is labelled by a red asterisk and at LN level by a green asterisk. Traits that showed a signiﬁcant difference between two N levels are labelled by a black asterisk (*P < 0.05).

NUE-related traits were evaluated in field experiments across four environments, including plant biomass (GY, SY, and HI), plant N concentration (GNC, SNC, and NHI), and N efficiency (NUE, Nup, NupE, and NutE). Under both HN and LN conditions, the parental line Ye478 had higher GY and SY than those of parental line Wu312 (Fig. 1, Table 2, and Supplementary Table S2). Under LN conditions, GY of both lines decreased by 9.5% and SY by 13.6%, but the differences were not significant. Total N accumulation as indicated by Nup significantly decreased by up to 29.4% because N concentration in grain and stover of plants were decreased by up to 16.1% and 28.7%, respectively. Nup in Ye478 reduced 16.8% owing to LN stress, but by almost double that (29.4%) in Wu312, suggesting a lower sensitivity of Ye478 to N deficiency (Supplementary Table S2). Importantly, Ye478 had ~32–37% higher NUE than that of Wu312 under both N conditions. Of the two main components of NUE, NupE was 25–33% higher in Ye478, but NutE did not significantly differ between the two genotypes (Fig. 1, Table 2, and Supplementary Table S2).

**Table 2.** Statistics for phenotypes of RIL population for NUE-related traits evaluated in the field and for RSA-related traits evaluated in hydroponics under HN and LN levels

Category	Trait	HN						LN						% of reduction mean^b^
		Parent		RILs				Parent		RILs				
		Ye478	Wu312	Range	mean	CV (%)	*h* ^*2*^ (%)^a^	Ye478	Wu312	Range	mean	CV (%)	*h* ^*2*^(%)	
NUE- related traits	GY	488.4	368.6*	57 –762	357	32.2	69.5	441.9	334.5*	40 –704	345	32.7	67.9	−3.4
	NUE	19.4	14.7*	5.0 –32.0	14.3	31.3	65.8	73.6	53.5*	21.0–110.0	57.2	30.2	67.7	–
	Nup	16.1	12.8*	5.0 –25.0	13.5	23.6	53.6	11.7	8.8*	4.0 –18.0	11.1	22.0	52.9	−18.2
	NupE	0.65	0.52*	0.17–0.99	0.55	22.6	48.0	1.68	1.27*	0.75–2.59	1.61	21.0	55.6	–
	NutE	35.9	34.9	10.7–53.5	30.6	25.3	75.8	47.7	45.2	16.2 –58.6	37.2	22.9	79.1	21.6
	SY	671.2	567.2*	260 –970	598	25.9	68.1	579.9	490.2*	254 –951	591	24.1	69.7	−1.2
	HI	0.46	0.44	0.15–0.54	0.38	22.8	68.0	0.48	0.45	0.09–0.53	0.38	22.6	73.1	0
	GNC	1.54	1.71*	1.3–1.9	1.6	8.6	66.1	1.29	1.49*	1.1–1.9	1.5	10.1	64.1	−6.3
	SNC	1.04	1.09	0.8–1.8	1.2	13.2	74.6	0.77	0.78	0.6–1.2	0.9	14.1	68.1	−25.0
	NHI	0.56	0.55	0.22–0.67	0.48	21.0	69.4	0.60	0.61	0.2–0.7	0.53	18.0	72.7	10.4
RSA- related traits	SRN	4.8	3.5*	2.5–6.2	3.7	14.8	68.8	4.7	3.4*	2.6–5.3	3.7	13.0	62.5	0
	CRN	6.3	5.4*	2.6–8.6	5.8	15.5	47.4	6.4	5.9	4.1–9.1	6.0	14.5	55.3	3.5
	LRN	47.2	32.6*	18.8–63.4	32.3	20.1	48.3	46.9	32.9*	19.0–48.1	32.4	19.2	52.3	0.3
	PRL	18.9	18.3	10.0–27.1	17.8	14.7	44.4	23.5	18.7*	12.3–30.5	19.3	16.8	36.4	8.2
	SRL	73.2	45.2*	32.9–118.5	73.9	21.0	57.7	87.5	49.5*	33.5–121.6	78.7	20.2	48.9	6.6
	CRL	46.9	45.2	167.0–91.1	41.2	24.9	38.9	57.8	50.9	22.2–113.0	51.9	26.2	36.5	26.1
	RDW	84.1	53.1*	36.7–139.5	70.7	24.1	53.6	107.7	62.4*	48.9–178.3	87.2	23.6	49.2	23.3
	SDW	300.8	171.7*	87.7–376.6	211.4	25.4	57.0	215.3	139.7*	82.9–313.9	167.5	24.3	58.3	−20.6
	R/S	0.27	0.29	0.24–0.54	0.34	15.2	52.9	0.48	0.43	0.35–0.85	0.52	14.2	60.5	52.9

^a^ *h* ^*2*^(%),broad-sense heritability; ^b^ % of reduction on mean in the RIL population = (LN − HN)/HN × 100%. Signiﬁcant difference between two genotypes is indicated by an asterisk (*P < 0.05). See Table 1 for an explanation of trait abbreviations and their units.

Within the RIL population generated by a cross of Ye478 and Wu312, the NUE-related traits were extensively segregated and normally distributed, and considerable phenotypic variation existed as revealed by the coefficient of variation (CV) ranging from 8.6% to 32.7% (Table 2). At LN level for RILs, average GY did not significantly decrease and Nup decreased by up to 18%. Accordingly, GNC and SNC were further decreased by up to 6.3% and 25%, respectively. For each NUE-related trait a significant correlation between HN and LN values was observed (Table S5). Besides genotype, significant effects were observed for N levels, environments, and their corresponding interactions, indicating strong G × E interaction (Supplementary Table S4). Nevertheless, the heritability (*h* ^*2*^) of NUE-related traits were rather high, varying from 48.0% to 75.8% under HN and from 52.9% to 73.1% under LN (Table 2).

As shown in Supplementary Figure S1A and Table S5, the phenotypic correlation between NupE and NUE was significant. The coefficients were higher under the LN condition (r = 0.52) than under the HN condition (r = 0.36). NutE had the higher correlation with NUE under both N levels (r = 0.57–0.62). As expected, NupE was highly related to plant biomass (GY and SY, r = 0.36–0.69), while NutE was highly positively correlated with HI for carbon and N (HI and NHI, r = 0.76–0.86) and negatively correlated with N concentration in plant tissue (SNC and GNC, r = 0.55–0.69).

### Evaluation of RSA-related traits in hydroponics

Three independent hydroponic experiments were used to evaluate different root traits under both HN and LN growth conditions (Fig. 1, Table 2, and Supplementary Table S3). The investigated RSA-related traits consisted of root biomass (RDW), root number (RN: SRN, CRN, LRN), and root length (RL: PRL, SRL, CRL). Irrespective of N levels, Ye478 had higher RN and RL than Wu312, as revealed by 42–45% more LRs, 62–77% more seminal roots, and 59–73% more RDW. Under LN stress, RL of Ye478 (PRL, SRL, and CRL) increased ~20%, twice as much as those of Wu312. RDW and R/S also increased more in Ye478. These results show that, compared with Wu312, Ye478 had a larger root system and showed a stronger root growth response to N deficiency (Fig. 1, Table 2, and Supplementary Table S3).

Within the RIL population, as for NUE-related traits, considerable phenotypic variation also existed for RSA-related traits (CV values ranged from 13.0% to 26.2%; Table 2). Each RSA-related trait significantly correlated under both HN and LN levels. The presence of strong environmental effects was revealed by the significant variance of G × E interaction (Table S4). Moreover, N deficiency for RILs resulted in increases in average RL (6.6–26.1%), RDW (23.3%), and R/S (52.9%), but little change in RN (0–3.5%). The heritability (*h* ^*2*^) of root-related traits were moderate, ranging from 38.9% to 68.8% under HN and from 36.4% to 62.5% under LN condition (Table 2).

As shown in Supplementary Figure S1B and Table S5, RDW was significantly correlated with RN and RL irrespective of N levels. Among the different root types, CR had the higher value of coefficients with RDW (r = 0.40–0.54). The RN and RL were correlated in the same root type with high coefficients (SR, r = 0.59–0.65; CR, r = 0.47–0.59). PRL also significantly associated with SRL and CRL (r = 0.24–0.55). Among all root traits, LRN showed no correlation with others, except a very small correlation with RDW (r = 0.11–0.24). Because it was counted within a 5cm region of the primary roots, LRN investigated here was more representative of the density of LRs. In spite of the stimulating effects on root growth, it is unlikely that LN stress affected the overall correlation among different root traits (Supplementary Figure S1B).

### Phenotypic relationship between RSA- and NUE-related traits

**Fig. 2.**
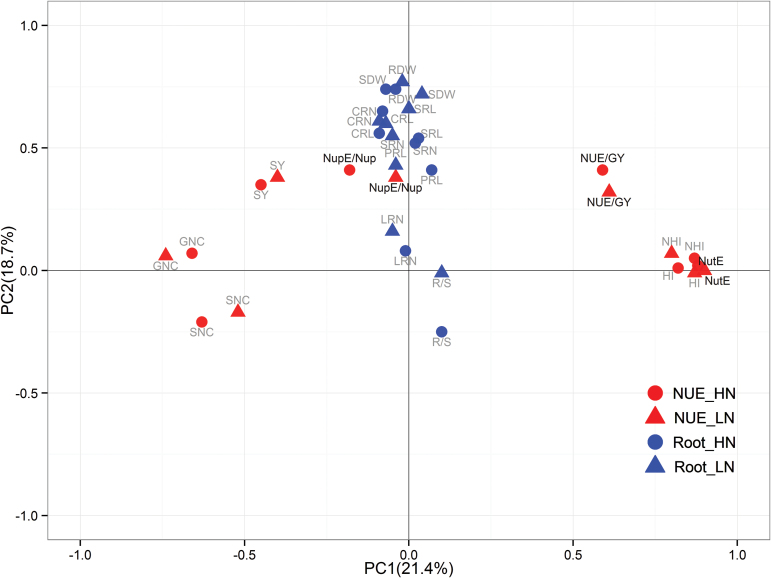
PCA of the RIL population for NUE-related traits (in red) evaluated in the field and for RSA-related traits (in blue) evaluated in hydroponics under the conditions of HN (circle) and LN (triangle) levels. Thirty-six traits were projected onto the first and second principal components.

To investigate the contribution of roots to plant N efficiency, the Pearson correlation between RSA- and NUE-related traits was determined, and a PCA performed to visualize the correlation (Table 3, Supplementary Table S5, Fig. 2). Overall, correlations between RSA- and NUE-related traits were much lower than those within RSA-related traits or NUE-related traits. Nevertheless, significant correlations were observed between some root traits and GY/NUE (r = 0.14–0.27), Nup/NupE (r = 0.15–0.31), and SY (r = 0.10–0.26). By contrast, no significant correlation was determined between most root traits and NutE, GNC, SNC, HI, or NHI (Table 3 and Supplementary Table S5). PCA demonstrated that root traits except LRN were most closely related with NupE under both HN and LN conditions (Fig. 2), indicating that NupE is more likely to be genetically associated with root traits. Irrespective of N levels, SRN and CRN appeared more related to NupE and NUE than other root traits (Table 3).The association between SRL and NupE was also relevant, and significantly greater under LN conditions with the highest coefficient value (r = 0.31). These results suggest that both SR and CR traits have a closer relationship with NUE and NupE than they do with NutE, and the relationship between SR and NupE was even strengthened under N-deficient conditions.

**Table 3.** Pearson’s correlation coefficients between RSA-related traits and NUE-related trait evaluated in the field and in hydroponics, respectively, under HN and LN levels

Treatment	Traits	HN							LN						
		SRN (217)^a^	CRN (217)	LRN (218)	PRL (218)	SRL (216)	CRL (217)	RDW (217)	SRN (214)	CRN (218)	LRN (218)	PRL (217)	SRL (207)	CRL (218)	RDW (218)
HN	NUE/GY (213)	0.18**	0.16*	0.00	0.16*	0.19**	0.09	0.14*	0.18**	0.17*	−0.02	0.12	0.27**	0.18**	0.16*
	NupE/Nup (208)	0.13	0.15*	−0.08	0.05	0.06	0.09	0.05	0.15*	0.12	−0.07	0.07	0.21**	0.12	0.05
	NutE (199)	0.00	0.04	−0.03	0.12	0.03	0.03	−0.01	−0.06	0.01	−0.03	0.06	0.04	0.03	0.01
LN	NUE/GY (216)	0.17*	0.05	0.05	0.07	0.11	−0.05	0.11	0.15*	0.10	0.04	0.09	0.20**	0.05	0.13
	NupE/Nup (207)	0.23**	0.13	-0.07	0.03	0.11	−0.04	0.03	0.31**	0.15*	−0.06	0.04	0.31**	0.02	0.05
	NutE (200)	−0.01	−0.09	0.02	0.07	0.01	−0.06	0.00	−0.04	−0.09	−0.01	0.03	0.09	−0.08	0.04

^a^ The number of points used to calculate the Pearson’s correlation coefficients. * Signiﬁcant at *P* < 0.05, ** Signiﬁcant at *P* < 0.01.

### Detection of QTLs for NUE- and RSA-related traits

**Fig. 3.**
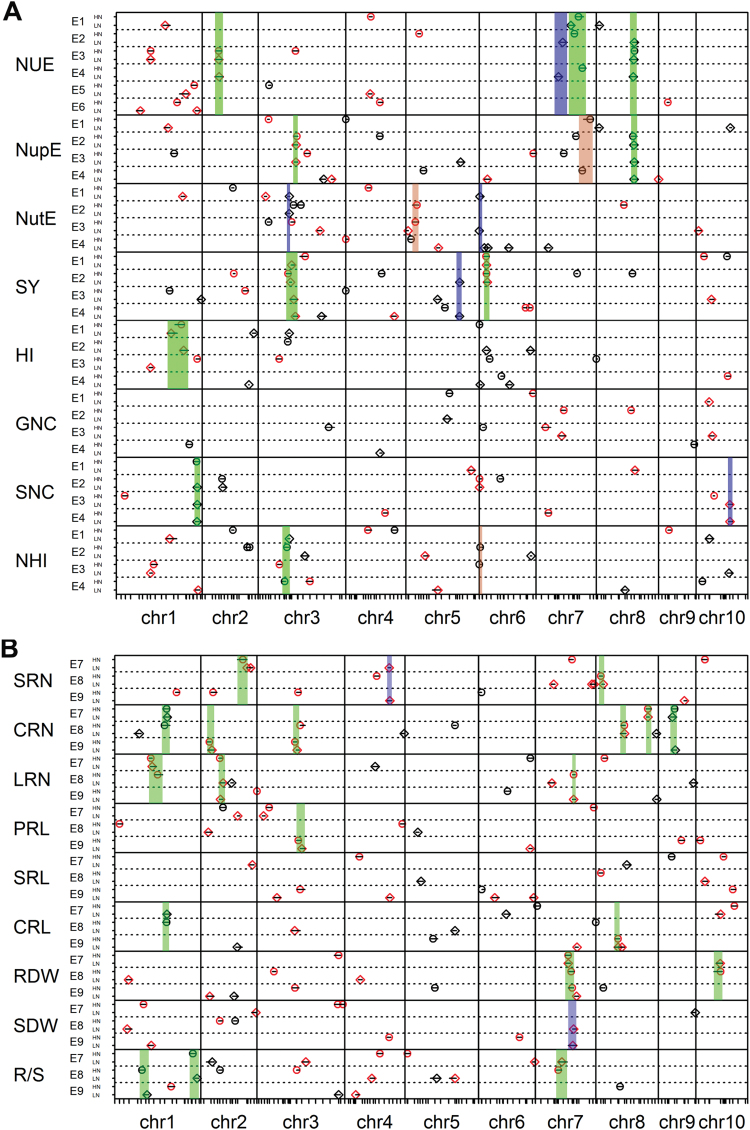
Genomic regions of 331 QTLs detected for **(A)** NUE-related traits across different field environments (E1–E6 for NUE, and E1–E4 for others) and **(B)** RSA-related traits across three independent hydroponics experiments (E7–E9) under HN (open circles) and LN (open diamonds) levels. For each QTL symbol, the horizontal line indicates conﬁdence intervals and the centre of the plot indicates the most likely position. Red symbolises a positive additive effect from Ye478 alleles and black a positive additive effect from Wu312 alleles. The rectangles represents the sQTL regions across the genome: green is representative for the constitutive sQTL, brown for HN-specific sQTL, and blue for the LN-specific sQTL.

In the RIL population, a total of 331 putative QTLs for all investigated traits through all independent experiments were identified across all 10 maize chromosomes, including 184 QTLs for NUE-related traits detected in the field and 147 QTLs for RSA-related traits detected in hydroponics (Fig. 3, Supplementary Table S6). Similar numbers of QTLs were detected under HN and LN conditions for both NUE-related traits (94 under HN, 90 under LN) and root-related traits (72 under HN, 75 under LN) (Supplementary Table S6). Within the identified QTLs for NUE, a similar proportion of QTLs carried the favourable allele that originated from either the parental line Ye478 or Wu312. By contrast, ~70% of identified QTLs for roots had the favourable alleles from the larger-rooted parent Ye478. Total phenotypic variation explained by the QTLs for each NUE-related trait ranged from 4.2% to 53.6%, and those for each root-related trait ranged from 4.7% to 29.7% (Supplementary Table S6).

Those QTLs repeatedly detected across the different environments were considered to be stable QTLs (sQTLs). sQTLs also had more than 1-LOD confidence interval overlapping and a positive additive effect from alleles of the same parental line (Supplementary Table S7). Constitutive sQTLs were defined for NUE-related traits if they were detected in at least three of four environments under both HN and LN conditions, and for RSA-related traits if they were detected in at least two of three independent hydroponics experiments. The QTLs that were exclusively detected in at least two environments under either HN or LN conditions were represented as HN-specific or LN-specific sQTLs. Thus, a total of 39 sQTLs were generated that contained 101 QTLs, about 30% of the total number of individual QTLs (Fig. 3 and Supplementary Table S7). NUE-related traits had 18 sQTLs, including 10 constitutive sQTLs, 3 HN-specific sQTLs and 5 LN-specific sQTLs (Fig. 3A and Supplementary Table S7). RSA-related traits had 21 sQTLs and most of them were constitutively expressed except for two LN-specific QTLs (Fig. 3B and Supplementary Table S7). Therefore, these sQTLs most likely contributed significantly to the genetic basis of NUE and RSA traits, and were relatively less influenced by the environments.

For NUE traits, a total of 29 significant QTLs were detected with LOD scores ranged from 2.6 to 6.8, and explained 5.8–19.0% of phenotypic variation (Fig. 3A and Supplementary Table S6). Of these QTLs, four sQTLs were found and only one constitutive sQTL at chromosomal region bin2.04 had an allelic effect from Ye478 (Supplementary Table S7). Twenty-six QTLs for NupE were identified that explained 7.1–35.4% of phenotypic variation. Twenty-five QTLs for NutE were identified that explained 8.0–40.5% phenotypic variation. An HN-specific and two LN-specific sQTLs were found from the NupE and NutE QTLs, respectively. By contrast, for the traits of tissue N concentration (GNC and SNC), relatively fewer QTLs (14 and 17) and only a few sQTLs were detected (Supplementary Tables S6 and S7).

For RSA traits, a total of 54 significant QTLs were associated with RN, included 18 for SRN, 19 for CRN, and 17 for LRN (Fig. 3B and Supplementary Table S6). Relatively fewer QTLs (44) were associated with RL traits, including 14 for PRL, 15 for SRL, and 15 for CRL. Most root QTLs had minor genetic effects because they all explained less than 20% of the phenotypic variation (Supplementary Table S6). More sQTLs for RN (12) were found than for RL (3), indicating that RN traits were relatively less influenced by the environments. Favourable alleles from Ye478 were most influential in QTLs for PR- and SR-related traits, but not for CR-related traits (Supplementary Table S6). A total of 16 and 13 QTLs were detected for RDW and SDW, respectively, which were largely driven by the alleles from Ye478. Other than those for NUE, most of the identified sQTLs for roots were constitutively expressed.

### Identification of QTL clusters for NUE- and RSA-related traits

**Fig. 4.**
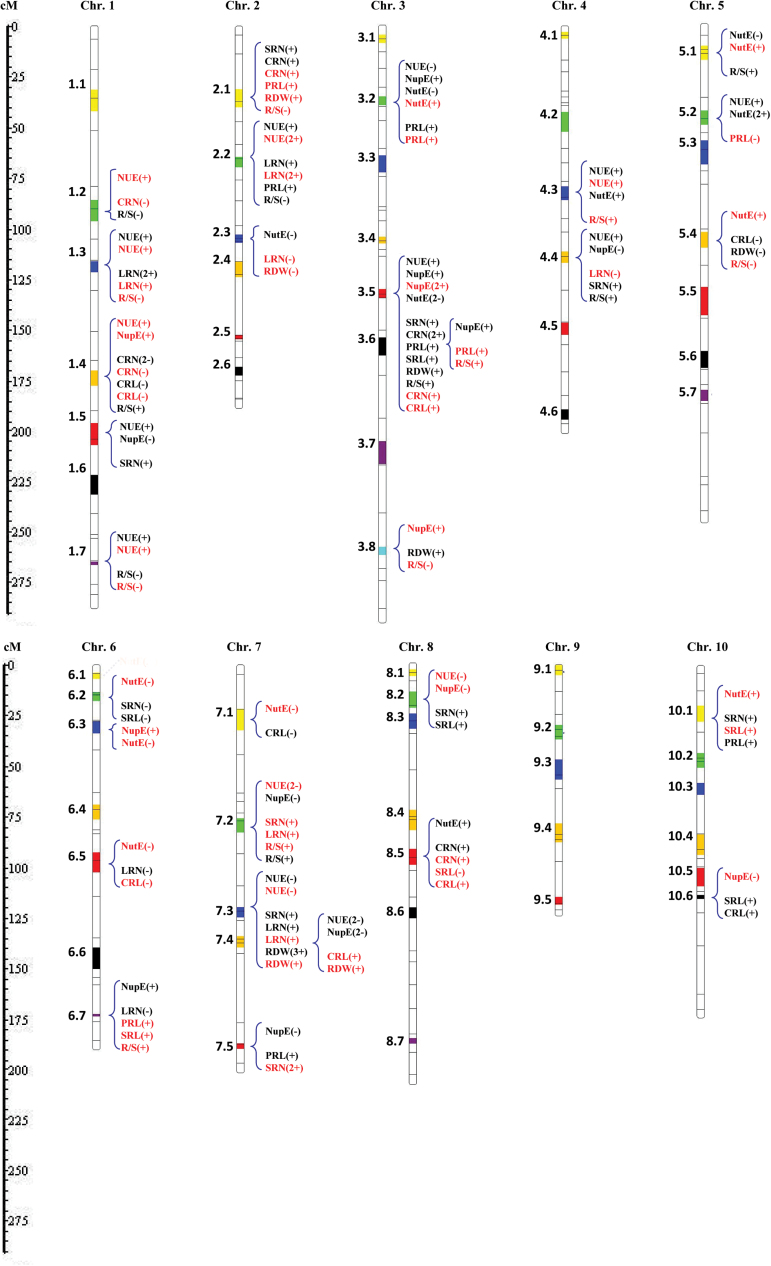
Location of the QTL clusters detected for all investigated traits as revealed by meta-QTL analysis. A vertical column represents each maize chromosome (chr) with the marker intervals where the QTL cluster was located. Each coloured boxplot represents the identified QTL cluster across all chromosomes. The name of each QTL cluster is give on the left of the chromosome. The QTLs contained within this cluster are given on the right of the chromosome. The names of the QTLs in the specific clusters are presented in which QTLs for NUE-related traits (NUE, NupE, and NutE) and QTLs for RSA-related traits (CRN, CRL, SRN, SRL, PRL, LRN, RDW, and R/S) coincided. Those QTLs detected at HN levels are indicated in black, and at LN levels in red. The number between parentheses indicates how many experiments this QTL was detected in. Additionally, a positive (+) and negative (−) sign represent positive additive effects from Ye478 and Wu312, respectively.

By a method of meta-analysis, the identified 331 QTLs for all investigated traits across all the environments were assigned into 64 distinct QTL clusters (Cl) (Fig. 4, Supplementary Figure S2 and Supplementary Table S8). Each QTL cluster consisted of 5.2 QTLs on average with a range of 1–17. The QTLs for NUE-related traits were grouped into 52 QTL clusters. Among them, 39 QTL clusters that contained QTLs for at least one trait of NUE, NupE, or NutE were defined as NE-QTL clusters. Nine NE-QTL clusters (Cl1.4, 1.5, 3.2, 3.5, 4.4, 7.2, 7.4, 8.2, and 8.6) had QTLs for both traits of NUE and NupE, and five NE-QTL clusters (Cl1.6, 3.2, 3.5, 4.3, and 5.2) for both traits of NUE and NutE (Supplementary Figure S3A and Supplementary Table S8). Two NE-QTL clusters (Cl3.2 and 3.5) at chromosome 3 simultaneously harboured QTLs associated with NUE, NutE, and NupE (Fig. 4 and Supplementary Figure S3A), and the QTLs for NupE were contributed to by alleles coming from Ye478 (Fig. 4 and Supplementary Table S7).

All 134 RSA-related QTLs except those for SDW were assigned into 48 QTL clusters that were defined as RSA-QTL clusters (Fig. 4 and Supplementary Table S8). More than half of QTLs for RN and RL presented as co-localized clusters (Supplementary Figure S3C). Two QTL cluster (Cl3.5 and 2.1) contained QTLs for RN, RL, and root biomass (Fig. 4 and Supplementary Figure S3C). By contrast, less than one-third of QTL clusters overlapped between each different root type (CR, SR, PR, and LR), and no common clusters for all root types was observed (Fig. 4 and Supplementary Figure S3D). Two of the largest QTL clusters for roots, Cl3.5 and Cl7.3, harboured up to nine and seven root-related QTLs at the chromosomal regions bin3.04 and bin7.03, respectively. Cl3.5 had QTLs for most root traits (RN, RL, and root biomass), while Cl7.3 had QTLs more specific for RN. Both QTL clusters had the favourable alleles coming from Ye478. In addition, Cl1.3 and Cl2.2 were the QTL clusters for the LR traits, and had favourable effects from alleles coming from Ye478. Cl1.4 had QTL clusters for CR traits with favourable alleles coming from Wu312 (Fig. 4).

### Determination of QTL clusters in which NE- and RSA-QTLs were co-localized

Of 39 NE-QTL cluster, 28 (~70%) were associated with RSA-QTL clusters and were defined as RSA-NE-QTL clusters (Fig. 4 and Supplementary Figure S3A). Among them, 15 QTL clusters overlapped between RSA and NUE traits, 13 between RSA and NupE, and 12 between RSA and NutE. Most common QTL clusters between NUE and NupE were associated with RSA-QTL clusters (Supplementary Figure S3A). More than 50% of RSA-QTLs expressed under both HN and LN condition co-localized with NE-QTL clusters (Supplementary Figure S3B). Additionally, 75% of HN- and 62.5% of LN-specific RSA-QTLs co-localized with NE-QTL clusters. Irrespective of RN, RL, or root biomass, more than 50% of QTL cluster co-localized with NE-QTL clusters (Supplementary Figure S3C). In terms of root types, ~50% of CR- and SR-related QTL clusters, and up to 60% of PR- and LR-QTL clusters, co-localized with those for NE traits (Supplementary Figure S3D).

The largest QTL cluster, Cl3.5, harboured QTLs for all traits of most root types (except LRN), and was also associated with QTLs for all NE traits (NUE, NupE, and NutE) under both HN and LN conditions (Fig. 4). Both Cl3.2 and Cl3.6 contained QTL clusters for the trait of PRL, and was associated with NupE under HN conditions. Cl1.4 for CR and Cl8.2 for SR traits co-localized with QTL clusters for both NUE and NupE detected under LN conditions. Cl1.3 and Cl2.2 contained QTLs mainly for LRN and co-localized with NUE QTL clusters. Cl6.7 harboured QTLs for NupE at HN conditions, and was associated with QTLs for PRL and SRL at LN levels. By contrast, a relatively smaller number of QTL clusters were found to be shared between the traits of RSA and NutE. Cl6.2 and Cl8.5 contained QTL clusters for SR and CR, respectively, which co-localized with QTL clusters for NutE (Fig. 4). Therefore, the presence of a considerable number of QTL clusters in which both NE- and RSA-QTLs were co-localized indicated the significant genetic contribution of RSA to NUE traits in maize.

### Performance of backcross lines containing RSA-NE-QTL clusters both *per se* and in hybrid combination

**Fig. 5.**
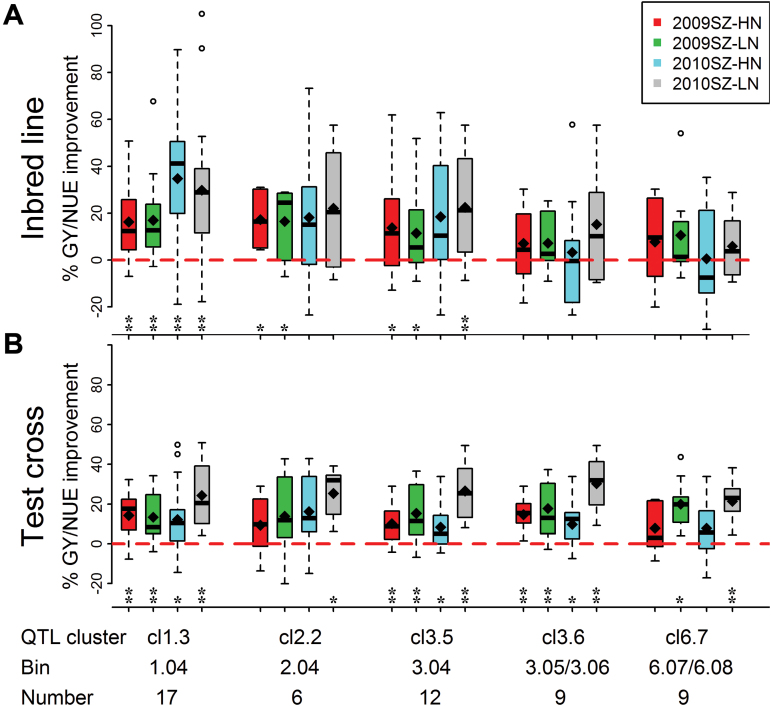
Validation of QTL cluster using the performance of an advanced backcross population at (A) *per se* and (B) testcross evaluation. The difference of GY and NUE (GY/NUE) between each ABL and the recurrent parent Wu312 was estimated by grain improvement (%),calculated as (ABL − Wu312)/Wu312. The diamond and black line within the boxplot indicates the mean value and the median value of the ABL, respectively. The bin indicates the genomic location of QTLs on the chromosome. The number of ABLs for each QTL cluster is also presented. A signiﬁcant difference within each environment tested by one-sample *t* test between the mean and 0 is indicated by an asterisk (*P < 0.05; **P < 0.01).

From a breeding point of view, five RSA-NE-QTL clusters (Cl1.3, 2.2, 3.5, 3.6, and 6.7) were considered because they had positive additive effect from the alleles coming from the large-rooted and high-NUE parent Ye478 (Fig. 5). Of these QTL regions, Cl3.6 at the chromosomal region bin3.05/3.06 and Cl6.7 at bin6.07/6.08 contributed positively to PRL and SRL traits, while both Cl1.3 at bin1.04 and Cl2.2 at bin2.04 most likely affected LRN. Cl3.5 at bin3.04 had a favourable effect on root systems in general. To evaluate the breeding value of RSA-NE-QTL clusters on the genetic improvement of GY/NUE, an ABL population (BC_4_F_3_) was generated by introgression of Ye478 genomic regions into the Wu312 background. The performance of the BC_4_F_3_ line *per se* and their testcrosses with line 178 were tested over two years under HN and LN levels (Fig. 5; Supplementary Table S9). Based on the flanked simple sequence repeat markers for Cl1.3, 2.2, 3.5, 3.6, and 6.7, the corresponding 17, 6, 12, 9, and 9 ABLs were selected (Fig. 5). These ABLs showed more than 90% genetic similarity to recurrent parent Wu312 (Supplementary Table S9). Compared to recurrent background Wu312, the GY/NUE of these ABLs showed apparent increases of 0.6–34.8% (mean, 13.8%) under HN and of 5.9–29.8% (mean, 15.9%) under LN conditions (Fig. 5A and Supplementary Table S9). At the hybrid level, the ABL testcross containing the targeted QTLs showed GY/NUE improvements of 7.8–16.2% (mean, 11.0%) under HN and of 13.4–30.2% (mean, 20.8%) under LN conditions (Fig. 5B and Supplementary Table S9). These results implicate the application of these QTL clusters as the targets for marker-assisted selection to improve GY/NUE in maize breeding programmes.

## Discussion

Given the important function of roots, plant breeders are turning their attention to roots to increase crop yield with the sustainable use of natural resources. Previous attempts to enhance phosphorus and water use efficiency have been achieved by genetic improvements of RSA in crops (Steele *et al.*, 2006; Landi *et al.*, 2010; Chin *et al.*, 2011). From a physiological perspective, selecting optimal RSA in breeding programmes has the potential to enhance N acquisition (Mackay and Barber 1986; Lynch *et al.*, 2013). However, this type of breeding practice is limited because of a lack of knowledge on genetic relationship between RSA and NUE and the corresponding genomic regions for targeted genetic manipulation. In this study, a maize RIL population was generated that possessed traits for both RSA and NUE to uncover the significant genetic associations between RSA and NUE, and to identify and validate several important underlying QTL clusters. These findings allow the establishment of an RSA-based approach for genetically improving NUE in maize.

### A RIL population for the genetic dissection of RSA- and NUE-related traits

The existence of valuable phenotypical variation for the targeted traits between the parental lines and within the corresponding genetic population allows the effective dissection of their genetic basis and identification of genomic regions for genetic improvements. In this study, the two parental lines, Y478 and Wu312, had contrasting phenotypes for both RSA and NUE traits (Fig. 1 and Supplementary Table S2 and S3). Compared to Wu312, Ye478 had the larger root system in hydroponics as well as the higher NupE in the field irrespective of HN and LN conditions. Previous studies on these two lines observed similar phenotypes at the seedling stage, and also suggested that the higher N uptake capacity in Ye478 relies on an advanced RSA performance and its response to N availability, rather than a higher N transport activity (Tian *et al.*, 2006; Liu *et al.*, 2009). At the lateral developmental stages (the silking and maturation stages) as revealed in the field, Ye478 also has the longer CR and LR length, and greater root biomass (Cai *et al.*, 2012; Peng *et al.*, 2010). For both RSA and NUE traits, the phenotypic variation within this RIL population is considerable larger, with modest to high heritability values (Table 2). As expected, QTL analysis revealed the additive effect of 70% of RSA-QTLs was derived from the donor allele of Ye478, given the genetic contribution of Ye478 to RSA traits (Supplementary Table S6). Consequently, this established RIL population from two contrasted lines in NUE and RSA was suitable for determining the relationship between RSA and NUE traits, and identifying favourable alleles for the RSA coming from Ye478 that can directly contribute to improving NupE.

### Genetic relationship between RSA and NUE traits in maize

The existence of a genetic relationship between RSA and NUE traits makes it essential for breeders to consider RSA as a selection criterion to improve NUE in maize breeding programmes. In this study, two lines of genetic evidence revealed the significant associations between RSA and NUE. First, the phenotypical correlation analysis and PCA showed that RSA significantly associated with NUE (r = 0.14–0.27) (Fig. 2 and Table 3). Importantly, RSA had a positive correlation with NupE (r = 0.15–0.31) but no correlation with NutE, suggesting that N acquisition, rather than N utilization, is most likely related to the function of roots. Among RSA traits, the contribution of SR and CR, in particular for length and number of SR under LN conditions, seems to be more relevant to NupE (r = 0.31) (Table 3). Besides bi-parental population, a similar correlation study using a diverse set of 74 inbred maize plants also showed that SR length of maize seedling plants was most correlated with GY under HN (r = 0.36) and LN (r = 0.24) levels (Abdel-Ghani *et al.*, 2013). Therefore, the selection of optimal root traits at the seedling stage is a promising approach to optimize maize NupE. Under field conditions, a significant correlation (r = 0.30–0.43) was also found between GY and RSA at an early development stage using the same advanced backcross population in this study (Cai *et al.*, 2012). Given that evaluation of maize RSA directly in the field is technically difficult, time- and labour-consuming, and strongly affected by the soil environments, plant breeders are likely to find high throughput, low-cost, and relatively stable analysis of RSA using hydroponic systems more acceptable for selection of root traits. Additionally, the significant relationships found between maize RSA and GY under water deficiency (r = 0.2–0.3; Tuberosa *et al.*, 2002) and phosphorus deficiency (r = 0.2–0.25, Zhu *et al.*, 2006) further implicate the essential function of RSA on adaptation of maize plants to abiotic stress in general. It is worth noting that under severe stress conditions the trade-off between root growth and above-ground growth leads to a negative correlation between roots and GY(Chen *et al.*, 2013).

Second, the coincidence of QTL clusters as revealed in this study further supports the genetic relationship between RSA and NUE traits (Fig. 4, Supplementary Figure S3A, and Supplementary Table S8). A large proportion of NE-related QTLs (70%) is associated with QTL clusters for RSA (Supplementary Figure S3A). Importantly, several QTL clusters contained QTLs for both RSA and NUE traits (Fig. 4). Most of these QTL clusters had favourable effects from alleles coming from the parent Ye478, which has a better root system than Wu312. Likewise, QTL analysis of another maize RIL population also revealed three QTL clusters for both N-uptake and root traits, and they also had favourable effect s from alleles coming from a parent with a superficial root system (Coque *et al.*, 2008). It is worth noting that the relationship between RSA and NupE traits at QTL clusters may correspond to control of pleiotropic genes or to different closely linked genes. Nevertheless, the presence of significant phenotypic correlation and co-localized common QTLs provide the solid genetic basis for establishing the association between RSA and NUE.

The AD is considered an important trait in maize, and often affects other physiological and agronomic traits directly or indirectly. In the present study, however, no phenotypic correlation was obtained between AD and NupE or RSA-related traits (Table S10). Furthermore, no QTLs for AD were found to co-localize with those of NupE or RSA-related QTLs, except one AD-QTL region for NutE and NHI traits (Table S11). Thus, these results indicate that the genetic variation of AD in this RIL population did not affect the phenotypic behaviour in terms of NupE or RSA traits.

### Five important QTL clusters for a root-based approach to improve NUE in maize

The coincidence of QTL clusters for RSA and NUE provides clues on their genetic association. More importantly, identification of the best alleles among these QTL clusters can help maize breeders implement high-NUE cultivars via a marker-assisted selection approach. In the present study, five important QTL clusters were identified in which QTLs for RSA and NUE coincided (Fig. 4). These QTL clusters contained root QTLs mainly for traits of RDW, LRN, PRL, and SRL, implicating that these root traits could be the targets for selection of high-NUE maize. In comparison to LR traits, the RDW, PRL, and SRL traits seem to be more promising as selection criteria because they are easily measured and less affected by the environment. Similarly, Abdel-Ghani *et al.* (2013) highlighted that the selection of RDW and SRL in maize seedling could lead to an increase in selection efficiency for GY of adult plants.

Of these important QTL clusters, the Cl2.2 cluster was localized on chromosome region bin2.04, and comprised NUE- and RSA-related traits (PRL and LRN). Tuberosa *et al.* (2003) discovered QTLs for root traits, i.e. brace root number and root pulling resistance, in a similar genomic region. A putative QTL, *root-ABA1*, that regulates both root size and GY also localized in this genomic region (Landi *et al.*, 2006). The largest QTL clusters located at chromosomal region bin3.04 were identified for RSA and NUE, in particular for NupE (Fig. 4). QTLs have previously been determined in this region for a large number of root traits (Qiu *et al.*, 2007; Liu *et al.*, 2008), as well as for yield-related traits under different environmental conditions (Agrama *et al.*, 1999; Messmer *et al.*, 2009; Qiu *et al.*, 2007). Coque *et al.* (2008) found a QTL for whole-plant N uptake at maturity (WpNup) in the genomic region bin6.07, which most likely co-localized with Cl6.7 in this study (Fig. 4). Collectively, these QTL clusters seems to be common for regulating maize RSA traits across different genetic backgrounds, and further involved in maize plants for improving water, nitrogen, and phosphorus use efficiency.

The QTLs with a significant impact on variation of NUE at hybrid level would have a greater breeding value, because hybrid varieties are often used by farmers. However, QTLs detected for NUE-related traits for *per se* value are different from the QTLs detected for testcross preformation (Coque and Gallais, 2008). In this study, the identified five important QTL clusters revealed a greater contribution to GY at both line *per se* and in testcross evaluation (Fig. 5 and Supplementary Table S9). Therefore, these alleles can confer a >20% gain of GY at hybrid level under LN stress conditions. Therefore, discovery of these QTLs opens a new and exciting opportunity for the manipulation of RSA via marker-assisted selection QTLs for improving NUE in maize.

## Conclusions

Despite the highly complex nature of NUE and RSA traits, this study shows the presence of genetic relationships and of major QTL clusters that coincide for both traits, implicating the potential genetic improvement for efficient N acquisition in maize. Where previous studies have investigated at the physiological level, the findings presented here provide a better knowledge of the genetic factors regulating RSA to produce N-efficient maize genotypes. From a breeding point of view, five identified QTL clusters, Cl1.3 (bin1.04), Cl2.2 (bin2.04), Cl3.5 (bin3.04), Cl3.6 (bin3.05/3.06), and Cl6.7 (bin6.07/6.08) have a positive effect on maize NUE at *per se* and hybrid levels, representing valuable targets for marker-assisted selection. Because it is impossible to distinguish a pleiotropy or linkage between close loci at the current level of QTL clustering, fine mapping and positional cloning are required to identify the underlying gene or genes to further promote root-based approaches to genetically improving maize NUE.

## Supplementary data

Supplementary data are available at *JXB* online

Supplementary File 1

Table S1. Summary of soil environment and fertilizer supply at the different environments (E1-E6).

Table S2. Statistics for NUE-related traits of the parent lines across six environments (E1-E6).

Table S3. Statistics for RSA-related traits of the parent lines across three independent experiments (E7-E9).

Table S4. ANOVA analysis for the traits across different environments and N levels.

Table S6. Main features of the QTLs detected for all investigated traits across all the environments.

Table S7. Summary of sQTLs for all investigated traits across all the environments.

Table S10. Pearson’s correlation coefficients between AD and NUE- or RSA-related traits under HN and LN levels.

Table S11. Detected QTLs for AD under HN and LN levels.

Figure S1. Network diagrams representing the phenotypic correlations between two traits within NUE-related traits and RSA-related traits based on their Pearson coefficients.

Figure S2. QTL clustering determined by MetaQTL software.

Figure S3. Numbers of QTL clusters for NUE- and RSA-related traits.

Supplementary File 2

Table S5. Pearson’s correlation coefficients (r) between all investigated traits of RILs grown under HN and LN levels.

Table S8. Summary of detected QTLs for all investigated traits in different environments and the identification of QTL clusters.

Table S9. The performance of inbred lines and testcrossed lines under HN and LN levels in 2009 and 2010.

## Funding

This work was supported by the Ministry of Science and Technology of China (2011CB100305, 2012AA100304); National Natural Science Foundation of China (31172015, 314211092); Danish Strategic Research Council (NUTRIE FFICIENT 10-093498); European Community the Seventh Framework Programme for Research (NUE-CROPS FP7-CP-IP 222645).

## Supplementary Material

Supplementary Data

## Abbreviations:

ABL: advanced-backcross line
AD: anthesis date
Cl: QTL clusters
CR: crown root
CRL: crown root length
CRN: crown root number
CP: Changping
CV: coefficient of variation
DBW: Dongbeiwang
GNC: grain nitrogen concentration
GY: grain yield
HI: harvest index
HN: high-nitrogen
LN: low nitrogen
LOD: logarithm of odds
LR: lateral root
LRN: lateral root number
N: nitrogen
NE: nitrogen efficiency
NHI: nitrogen harvest index
NUE: nitrogen use efficiency
Nup: nitrogen uptake
NupE: nitrogen uptake efficiency
NutE: nitrogen utilization efficiency
PCA: principal component analysis
PR: primary root
PRL: primary root length
QTL: quantitative trait loci
R/S: root to shoot ratio
RDW: root dry weight
RIL: recombination inbred line
RL: root length
RN: root number
RSA: root system architecture
SDW: shoot dry weight
SNC: stover nitrogen concentration
sQTL: stable QTL
SR: seminal root
SRL: seminal root length
SRN: seminal root number
SY: stover yield
SZ: Shangzhuang.
