# Supplementary material for: A genetic relationship between nitrogen use efficiency and seedling root traits in maize as revealed by QTL analysis
Source: J Exp Bot. 2015 Apr 6;66(11):3175–88. doi: 10.1093/jxb/erv127 (PMC4449538; doi:10.1093/jxb/erv127)
Supplement: Supplementary Data [file supp_66_11_3175__index.html]

A genetic relationship between nitrogen use efficiency and seedling root traits in maize as revealed by QTL analysis — A genetic relationship between nitrogen use efficiency and seedling root traits in maize as revealed by QTL analysis — Supplementary Data 

# A genetic relationship between nitrogen use efficiency and seedling root traits in maize as revealed by QTL analysis

## Supplementary Data

Data files

**Files in this Data Supplement:**

- Supplementary Data - Supplementary Data
- Supplementary Data - Supplementary Data
