# Supplementary material for: A genetic relationship between nitrogen use efficiency and seedling root traits in maize as revealed by QTL analysis
Source: J Exp Bot. 2015 Apr 6;66(11):3175–88. doi: 10.1093/jxb/erv127 (PMC4449538; doi:10.1093/jxb/erv127)
Supplement: Supplementary Data [file supp_erv127_jexbot141853_file001.pdf]

Journal of Experimental Botany

A genetic relationship between nitrogen use efficiency and seedling root traits in maize as revealed by QTL analysis

Pengcheng Li, Fanjun Chen, Hongguang Cai, Jianchao Liu, Qingchun Pan, Zhigang Liu, Riliang Gu, Guohua Mi, Fusuo Zhang, and Lixing Yuan

Supplementary File 1

Table S1. Summary of soil environment and fertilizer supply at the different environments (E1-E6).

Table S2. Statistics for NUE related traits of the parent lines across six environments (E1-E6).

Table S3. Statistics for RSA-related traits of the parent lines across three independent experiments (E7-E9).

Table S4. ANOVA for the traits across different environment N level trials.

Table S6. Main features of the QTLs detected for all investigated traits across all the environments.

Table S7. Summary of stable QTLs (sQTLs) for all investigated traits across all the environments.

Table S10. Pearson's correlation coefficients between anthesis date (AD) and NUE- or RSA-related traits under high N (HN) and low N (LN) levels.

Table S11. Detected QTLs for anthesis date (AD) under high N (HN) and low N (LN) levels.

Figure S1. Network diagrams representing the phenotypic correlations between two traits within NUE-related traits and RSA-related traits based on their Pearson coefficients.

Figure S2. QTL clustering determined by MetaQTL software.

Figure S3. Numbers of QTL clusters for NUE- and RSA-related traits.

Table S1. Summary of soil environment and fertilizer supply at the different environments (E1-E6)

Environment abbreviation	Location	Treatment[1]	Soil before plating					Fertilizer			Total[5] N Supply (kg/ha)
			Organic matter (g/kg)	total nitrogen (mg/kg)	Available Phosphorus[2] (mg/kg)	Available Potassium[3] (mg/kg)	Nmin[4] (kg/ha)	N (kg/ha)	P_2O_5 (kg/ha)	K_2O (kg/ha)	
E1	2006DBW	HN	23.0	1.0	17.0	157.5	73.1	180	120	80	253
		LN	-	-	-	-	73.1	0	120	80	73
E2	2007DBW	HN	-	-	-	-	160	180	120	80	340
		LN	-	-	-	-	84.0	0	120	80	84
E3	2007CP	HN	15.4	0.69	4.4	140.3	95.6	135	67.5	80	230
		LN	13.9	0.61	3.7	124.8	50.1	0	67.5	80	50
E4	2008CP	HN	-	-	-	-	72.1	135	67.5	80	207
		LN	-	-	-	-	67.1	0	67.5	80	67
E5	2009SZ	HN	15.8	0.83	26.7	103.8	109.0	180	120	80	289
		LN	-	-	-	-	52.6	0	120	80	52
E6	2010SZ	HN	12.7	0.73	28.3	97.6	81.0	180	120	80	261
		LN	12.5	0.77	29.0	141.0	37.0	0	120	80	37

[1] HN, high-nitrogen level; LN, low-nitrogen level.

[2] Available phosphorus was measured as Olsen-P.

[3] Available potassium was measured as exchangeable-K.

[4] Nmin was obtained from the analysis of inorganic N concentration (NH^{4+}-N and NO_3^--N) from fresh soils that were sampled as 30-cm-deep soil cores. Soil samples were collected from the field before planting.

[5] Total nitrogen supply were calculated as the sum of soils N (Nmin) and fertilizer N.

Table S2. Statistics for NUE related traits of the parent lines across six environments (E1-E6). Statistics for grain yield (GY), nitrogen use efficiency (NUE), nitrogen uptake (Nup), nitrogen uptake efficiency (NupE), nitrogen utilization efficiency (NutE), stover yield (SY), havest index (HI), grain nitrogen concentration (GNC), stover nitrogen concentration (SNC), nitrogen harvest index (NHI) of the parent lines grown in the filed under high-nitrogen (HN) and low-nitrogen (LN) levels across six environments

Trait	Units	Treatment	E1		E2		E3		E4		E5		E6		Lsmean[1]		% of Reduction[2]	
			Ye478	Wu312	Ye478	Wu312	Ye478	Wu312	Ye478	Wu312	Ye478	Wu312	Ye478	Wu312	Ye478	Wu312	Ye478	Wu312
GY	$g\ m^{-2}$	HN	428.1a[3]	365.6a*[4]	628.0a	451.0a*	597.5a	438.3a*	646.0a	526.4a*	475.8a	287.1a*	154.9a	143.1a	488.4	368.6	-9.5	-9.3
		LN	372.5b	339.6a*	538.7b	447.0a*	550.0a	318.8b*	633.0a	483.1a*	414.0a	280.4a*	143.4a	137.8a	441.9	334.5		
NUE	g/g	HN	16.9	14.4*	18.5	13.3*	25.9	19.0*	32.0	26.0*	16.5	9.9*	5.9	5.5	19.4	14.7	—	—
		LN	51.0	46.5*	64.1	53.2*	109.8	63.6*	96.2	72.1*	78.7	53.3*	38.7	37.3	73.6	53.5		
Nup	$g\ m^{-2}$	HN	12.6a	12.4a	19.9a	17.7a*	13.9a	10.9a*	17.6a	15.0a*	—[5]	—	—	—	16.0	14.0	-16.8	-29.4
		LN	10.0a	8.7b	14.5b	14.2b*	8.5b	6.3b*	12.2b	10.3b*	—	—	—	—	11.3	9.9		
NupE	g/g	HN	0.51	0.47	0.58	0.41*	0.63	0.52	0.86	0.67*	—	—	—	—	0.65	0.52	—	—
		LN	1.61	1.35*	1.77	1.24*	1.71	1.30*	1.74	1.18*	—	—	—	—	1.68	1.27		
NutE	g/g	HN	33.0a	30.7a*	32.0b	33.1a	41.6b	36.9b	37.2b	38.9a	—	—	—	—	35.9	34.9	32.5	29.5
		LN	33.7a	32.5a	36.6a	42.7a	64.4a	49.2a*	55.8a	56.4a	—	—	—	—	47.7	45.2		
SY	$g\ m^{-2}$	HN	596.1a	582.7a	780.7a	684.7*	546.9a	457.0a*	761.0a	544.5a*	—	—	—	—	671.2	567.2	-13.6	-13.6
		LN	588.0a	440.7b*	670.5b	588.8*	430.9b	412.7a	630.0b	518.7a*	—	—	—	—	579.9	490.2		
HI	g/g	HN	0.42a	0.39a	0.45a	0.40a	0.52a	0.49a	0.46a	0.49a	—	—	—	—	0.46	0.44	2.70	1.13
		LN	0.39b	0.44a	0.45a	0.43a	0.56a	0.44a*	0.50b	0.48a	—	—	—	—	0.48	0.45		
GNC	%	HN	1.42a	1.63a*	1.66a	1.82a*	1.54a	1.65a	1.53a	1.75a*	—	—	—	—	1.54	1.71	-16.1	-13.1
		LN	1.24b	1.46b*	1.53b	1.71b*	1.14b	1.34b*	1.25b	1.44b*	—	—	—	—	1.29	1.49		
SNC	%	HN	1.09a	1.10a	1.21a	1.38a*	0.85a	0.81a	1.02a	1.07a	—	—	—	—	1.04	1.09	-26.6	-28.7
		LN	0.92a	0.84b	0.94b	1.12b	0.52b	0.50b	0.68b	0.65b	—	—	—	—	0.77	0.78		
NHI	$g\ m^{-2}/g\ m^{-2}$	HN	0.48a	0.48a	0.52a	0.46a	0.66a	0.66a	0.56a	0.61a	—	—	—	—	0.56	0.55	7.71	11.69
		LN	0.46a	0.57b	0.57a	0.54a	0.74a	0.67a*	0.65a	0.67a	—	—	—	—	0.60	0.61		

[1] Least-squares means was calculated as described in Material and Methods; [2] % of Reduction on Mean = (LN-HN)/HN×100%; [3] Significant difference between treatments in the same environment was indicated by different letters (P<0.05); [4] Significant difference between two genotypes in the same environment was indicated by an asterisk (*, P<0.05); [5] Not available data was indicated by a dash (-).

Note: the data for grain yield (GY) and stover yield (SY) under high-N levels (HN) at E1 and E2 were obtained from Liu *et al*. (2011); the data for grain yield (GY) under low-N levels (LN) at E3 and E4 were obtained from Cai *et al.* (2012).

Table S3. Statistics for RSA-related traits of the parent lines across three independent experiments (E7-E9). Statistics for seminal root number (SRN), crown root number (CRN), lateral root number (LRN), primary root length (PRL), seminal root length (SRL), crown root length (CRL), root dry weight (RDW), shoot dry weight (SDW) and root to shoot ratio (R/S) of the parent lines grown in hydroponics under high-nitrogen (HN) and low-nitrogen (LN) levels across three independent experiments.

Trait	Units	Treatment	E7		E8		E9		Lsmean[1]		% of Reduction[2]	
			Ye478	Wu312	Ye478	Wu312	Ye478	Wu312	Ye478	Wu312	Ye478	Wu312
SRN	number	HN	4.9a	3.5a	4.7a[3]	3.3a	4.9a	3.7a	4.8	3.5	-2.08	-4.29
		LN	4.7a	3.4a	4.6a	3.4a	4.8a	3.3a	4.7	3.4		
CRN	number	HN	8.4a	6.9a	7.3a	6.4a	5.2a	4.3a	6.3	5.4	1.60	11.21
		LN	8.1a	7.9a	7.4a	7.3a	5.3a	4.6a	6.4	5.9		
LRN	number	HN	31.2a	21.4a*[4]	49a	35.8a*	45.3a	29.3a*	47.2	32.6	-0.53	0.92
		LN	31.7a	21.6a*	47.5a	36a*	46.3a	29.7a*	46.9	32.9		
PRL	cm	HN	17.4a	15.9a	18a	19.8a	19.9a	16.7a	18.9	18.3	24.01	2.19
		LN	20.6a	15.6a	20.7a	20.3a	26.3b	17a*	23.5	18.7		
SRL	cm	HN	129.7a	91.2a*	67.9a	50.4a*	78.5a	40a*	73.2	45.2	19.54	9.51
		LN	125.9a	107.4a*	75b	53.8a*	100b	45.2a*	87.5	49.5		
CRL	cm	HN	70.5a	67.3a	47.3a	48.2a	46.4a	42.2a	46.9	45.2	23.27	12.72
		LN	78.2a	85.2b	56.4b	51.2b	59.1b	50.7b	57.8	50.9		
RDW	mg $plant^{-1}$	HN	127.5a	74.9a*	120a	76.7a*	48.2a	29.4a*	84.1	53.1	28.00	17.53
		LN	157.7b	94.4b*	145.9b	86.4b*	69.4b	38.3b*	107.7	62.4		
SDW	mg $plant^{-1}$	HN	360.1a	204.6a*	321a	202.9a*	280.5a	140.5a*	300.8	171.7	-28.43	-18.67
		LN	302.3b	192.3b*	244.2b	177.6b*	186.3b	101.7b*	215.3	139.7		
R/S	mg/mg	HN	0.35a	0.37a	0.37a	0.38a	0.17a	0.21a	0.27	0.29	77.78	48.28
		LN	0.52b	0.49b	0.60b	0.49b*	0.37b	0.38b	0.48	0.43		

[1] Least-squares means was calculated as described in Material and Methods; [2] % of Reduction on Mean = (LN-HN)/HN$\times$100%; [3] Significant difference between treatments in the same environment is indicated by different letters ($P<0.05$); [4] Significant difference between two genotypes in the same environment is indicated by an asterisk (*, $P<0.05$).

Table S4 ANOVA analysis for the traits across six environments and two N levels.

Trait	env	N level	env × N level	line	env×line	N level ×line	env× N level ×line	Error
GY	11556035.4**	179.8	466048.4**	202961.2**	65861.8**	18126.1**	18123.7**	4734.1
NUE	106305.2**	2101895.4**	28807.7**	2077.0	794.6**	706.1**	397.2**	64.9
Nup	2165.0**	6183.5**	1304.0**	108.0**	48.0**	15.7**	17.6**	4.5
NupE	33.32**	1074.03**	5.23**	0.84**	0.41**	0.25**	0.19**	0.05
NutE	38749.4**	48423.4**	6660.7**	939.6**	198.6**	102.4**	82.8**	27.4
SY	9029658.1**	455073.3**	376181.3**	452243.0**	116958.8**	39893.1**	44525.9**	10178.4
NHI	8.825**	3.461**	0.117**	0.1294**	0.0304**	0.0154**	0.014**	0.005
HI	1.973**	0.127**	0.003	0.090**	0.020**	0.007**	0.007**	0.003
SNC	48.94**	72.89**	3.37**	0.32**	0.06**	0.07**	0.04**	0.01
GNC	29.38**	9.29**	9.50**	0.36**	0.09**	0.04**	0.04**	0.01
SRN	85.4**	0.1	1.4	4.0**	1.0**	0.6**	0.6**	0.5
CRN	2362.4**	31.4**	12.5**	11.3**	5.1**	1.6**	1.5**	1.1
LRN	63709.6**	0.4	249.6	507.9**	221.1**	76.2**	80.5**	38.5
PRL	1335.2**	1750.4**	891.2**	101.5**	51.4**	32.8**	33.6**	15.7
SRL	682679.4**	29958.2**	10443.9**	3290.5**	1305.4**	767.0**	709.8**	272.2
CRL	123239.3**	89008.2**	8200.1**	1611.5**	967.7**	360.9**	369.5**	98.7
RDW	1261921.8**	254539.9**	11573.6**	4945.4**	2272.6**	542.0**	560.4**	230.8
SDW	1262474.3**	1803856.7**	73596**	32210.2**	12948.7**	3609.9**	3134.6**	1131.9
R/S	16.747**	26.756**	0.591**	0.043**	0.016**	0.011**	0.009**	0.003

Note: The numbers indicate the mean square value. Significant level at $P<0.01$ was indicated by **.

Table S6. Main features of the QTLs detected for all investigated traits across all the environments

Category	Trait	treatment [1]	Number	LOD	$R^{2, 2}$	ADD [3]	
						(+)	(-)
NUE-related QTL	NUE	HN	14	2.7-6.8	6.4-19.0	9	5
		LN	15	2.6-4.3	5.8-13.3	8	7
	NupE	HN	13	2.7-5.5	7.2-35.4	4	9
		LN	13	2.5-4.7	7.1-31.4	6	7
	NutE	HN	11	2.6-4.1	8.8-36.6	6	5
		LN	14	2.6-4.3	8.0-40.5	6	8
	SY	HN	16	2.5-4.3	4.2-37.0	9	7
		LN	13	2.5-4.2	5.1-19.0	8	5
	HI	HN	9	2.5-3.0	6.4-19.6	4	5
		LN	10	2.6-3.9	5.8-19.9	3	7
	GNC	HN	9	2.5-3.9	4.7-15.7	4	5
		LN	5	2.6-3.7	5.9-39.6	3	2
	SNC	HN	8	2.9-3.6	5.9-53.6	5	3
		LN	9	2.5-3.5	6.1-20.1	5	4
	NHI	HN	14	2.5-3.3	6.2-19.2	5	9
		LN	11	2.6-5.7	7.6-47.5	5	6
	Total	HN	94	2.5-6.8	4.2-53.6	46	48
		LN	90	2.5-5.7	5.1-47.5	44	46
Root-related QTL	SRN	HN	9	2.6-4.5	5.2-19.8	8	1
		LN	9	2.9-4.8	6.0-12.4	9	0
	CRN	HN	9	2.6-3.8	5.5-17.7	5	4
		LN	10	2.6-3.8	6.2-25.2	4	6
	LRN	HN	8	2.6-6.3	5.5-23.7	6	2
		LN	9	2.5-4.1	4.8-15.6	5	4
	PRL	HN	8	2.6-4.0	5.3-17.0	7	1
		LN	6	2.9-5.4	6.8-29.7	5	1
	SRL	HN	7	2.5-4.6	4.8-18.5	5	2
		LN	8	2.6-4.9	6.1-12.3	6	2
	CRL	HN	6	2.5-3.2	5.0-20.3	2	4
		LN	9	2.5-3.6	5.1-24.8	5	4
	RDW	HN	9	2.5-3.2	4.7-9.1	7	2
		LN	7	2.5-4.5	5.4-23.7	6	1
	SDW	HN	7	2.5-3.6	5.3-12.5	6	1
		LN	6	2.5-3.2	5.6-14.7	5	1
	R/S	HN	9	2.6-4.5	5.9-15.5	5	4
		LN	11	2.5-3.4	5.4-17.4	6	5
	Total	HN	72	2.5-6.3	4.7-23.7	51	21
		LN	75	2.5-5.4	4.8-29.7	51	24

[1] HN and LN presented high-nitrogen and low-nitrogen levels, respectively.
[2] The squared partial correlation coefficient that is the coefficient of determination between the respective QTL and the phenotypic observation.
[3] A positive and negative values represented Ye478 and Wu312, respectively, which carried the allele for an increase of trait value.

Table S7. Summary of stable QTLs (sQTLs) for all investigated traits across all the environments

Categ.	Trait	QTL	Environment		Bin [1]	Cluster	Position	LOD	$R^{2,2}$	Add.[3]
			HN	LN						
NUE-related QTL	NUE	NUE2	E3	E3,E4	2.04	2.2	55.0-56.0	2.9-3.2	5.8-7.8	2.45 to 8.58
		NUE7-1	-	E2,E4	7.02	7.2	74.0-87.9	2.7-4.2	5.9-13.3	-5.91 to -9.25
		NUE7-2	E1,E2, E4	E1	7.03/7.04	7.3/7.4	115.0-153.0	2.8-4.6	6.4-12.4	-1.20 to -5.08
		NUE8	E3	E2,E3,E4	8.07	8.6	122.0-126.0	3.0-6.8	7.2-13.6	-3.66 to -8.56
	NupE	NupE3	E2	E2, E3	3.04	3.5	124.5-127.5	2.7-4.0	10.2-21.5	4.38 to 22.59
		NupE7	E1,E4	-	7.04/7.05	7.4/7.5	152.7-179.1	2.7-3.3	7.2-35.4	-2.77 to -12.72
		NupE8	E2	E2,E3, E4	8.07	8.6	120.9-125.4	2.6-4.7	7.1-10.3	-4.28 to -16.75
	NutE	NutE3	-	E1,E2	3.04	3.4	102.6-102.7	3.3-4.3	8.0-12.0	-2.61 to -3.46
		NutE5	E2,E3	-	5.01	5.2	31.7-36.7	2.6-2.7	9.7-9.8	2.61 to 3.21
		NutE6	-	E1,E3	6.00	6.1	0.0-2.0	2.6-2.7	8.0-9.4	-2.08 to -3.30
	SY	SY3	E2	E1,E2, E3,E4	3.04	3.4/3.5	98.6-122.5	2.5-4.2	5.1-18.6	30.16 to 102.17
		SY5	-	E2,E4	5.06	5.7	177.0-177.8	2.8-3.5	5.1-8.2	-45.9 to -67.4
		SY6	E1, E2	E1,E2	6.02	6.3	22.9-26.8	2.9-3.8	4.9-10.0	37.27 to 63.67
	HI	HI1	E1	E1,E2	1.07	1.4/1.6	180.5-221.5	2.7-3.4	18.2-19.9	0.05
	SNC	SNC1	E1	E2,E3,E4	1.10	1.7	264.1-266.5	2.5-3.6	6.1-8.5	-0.04 to -0.05
		SNC10	-	E3, E4	10.04	10.6	110.8-111.8	2.9-3.5	6.5-8.4	0.04
	NHI	NHI3	E2,E4	E1	3.04	3.4	87.2-102.7	2.7-3.4	8.5-9.1	-0.03 to -0.04
		NHI6	E2,E3	-	6.00	6.1	0.0-3.0	2.9-3.1	9.0-9.9	-0.04
Root-related QTL	SRN	SRN2	E7	E7	2.06/2.07	2.5	138.6-152.6	2.6-3.0	6.2-19.8	0.16 to 0.31
		SRN4	-	E7,E9	4.07	4.5	145.9-147.9	3.0-3.1	6.0-7.4	0.17 to 0.19
		SRN8	E8	E8	8.02/8.03	8.2/8.3	16.7-24.6	3.2-4.5	6.5-9.1	0.15
	CRN	CRN1	E7,E8	E7	1.06/1.07	1.4	163.5-172.5	2.6-3.1	5.7-25.2	-0.27 to -0.66
		CRN2	E9	E9	2.02	2.1	28.6-36.8	2.8-3.0	6.4-14.3	0.26 to 0.41
		CRN3	E9	E9	3.04	3.5	125.5-132.7	3.2-3.7	7.8-14.7	0.29 to 0.45
		CRN8-1	E8	E8	8.06	8.5	94.3-94.7	2.8-3.2	5.8-6.4	0.25 to 0.28
		CRN8-2	E7	E7	8.08	8.7	172.0	3.0-3.2	5.9-6.2	0.38 to 0.44
		CRN9	E7	E7 E9	9.02	9.3	46.2-56.1	2.8-3.8	5.5-17.4	-0.26 to -0.53
	LRN	LRN1	E7,E8	E7	1.04/1.05	1.3	118.6-140.9	2.6-6.3	13.2-23.7	3.13 to 3.87

Table S7 continued

Categ.	Trait	QTL	Environment		Bin[1]	Cluster	Position	LOD	$R^{2,2}$	Add.[3]
			HN	LN						
Root-related QTL	LRN	LRN2	E7	E8,E9	2.04	2.2	62.8-73.6	2.5-3.4	4.8-6.7	1.97 to 2.67
		LRN7	E8	E9	7.03	7.3	125.9	3.0-4.1	5.5-8.1	1.82 to 2.73
	PRL	PRL3	E9	E9	3.04/3.05	3.5/3.6	136.7-148.1	3.5	6.8-11.3	1.28 to 1.84
	CRL	CRL1	E8	E7	1.06	1.4	170.5-171.5	3.2-3.6	20.3-24.8	-5.03 to -13.22
		CRL8	E9	E9	8.05	8.4	73.7	2.8-2.9	5.7-5.9	3.80 to 4.15
	RDW	RDW7	E7,E8, E9	E7	7.03	7.3	108.0-121.1	2.5-3.2	4.7-7.7	2.79 to 10.61
		RDW10	E8	E7	10.03	10.4	79.7-81.6	2.6-2.8	5.3-8.5	5.25 to 11.79
	SDW	SDW7	-	E8, E9	7.03	7.3	123.1-125.9	2.7-2.9	6.1-6.2	9.23-12.52
	R/S	R/S1-1	E8	E9	1.03	1.2/1.3	89.3-105.8	2.6-3.1	8.7-8.8	-0.02 to -0.03
		R/S1-2	E7	E8	1.08/1.10	1.7	257.1-270.5	2.6-2.8	8.1-9.6	-0.02 to -0.04
		R/S7	E8	E7	7.02	7.2	74.9-86.9	2.5-2.7	6.1-8.6	0.02 to 0.03

[1] Chromosome bins of the marker and position taken from IBM 2008.
[2] The squared partial correlation coefficient that is the coefficient of determination between the respective QTL and the phenotypic observation.
[3] A positive and negative values represented Ye478 and Wu312, respectively, which carried the allele for an increase of trait value.

Table S10. Pearson's correlation coefficients between anthesis date (AD)[a] and NUE- or RSA-related traits under high N (HN) and low N (LN) levels.

Trait	GY/NUE	Nup/NupE	NutE	SY	HI	GNC	SNC	NHI	SRN	CRN	LRN	PRL	SRL	CRL	RDW	SDW	R/S
AD-HN	-0.35*	0.04	-0.30*	0.16*	-0.28*	0.27*	0.20*	-0.44*	-0.03	-0.06	-0.03	-0.08	-0.09	-0.06	-0.14*	-0.11	0.08
AD-LN	-0.24*	-0.16	-0.38*	0.21*	-0.54*	0.41*	0.10	-0.45*	-0.11	0.05	0.13	0.01	-0.02	0.04	-0.05	-0.05	0.06

[a] Data for AD came from the experiment at E4 (Changping, CP, in 2008) as described by *Cai et al., 2011, Molecular Breeding*.

* Significant at $P < 0.05$.

Table S11. Detected QTLs for anthesis date (AD)[a] under high N (HN) and low N (LN) levels.

Trait	Environment	Treatment	Chr.	Position	Flanking markers		LOD	Additive[b]	R^{2c}	NUE/RSA traits[d]
AD	E4	LN	1	177	umc1335	bnlg1556	2.5	-0.9	5.3	—
AD	E4	LN	1	199	bnlg1556	bnlg1025	3.0	-1.2	6.2	—
AD	E4	HN	1	202	bnlg1556	bnlg1025	3.1	-0.9	6.4	—
AD	E4	HN	2	115	nc003	umc1637	4.8	1	9.8	—
AD	E4	LN	2	115	nc003	umc1637	4.5	0.9	9.2	—
AD	E4	HN	4	50	phi295450	umc1294	3.2	1.2	6.7	—
AD	E4	HN	5	98	bnlg2323	umc1332	3.2	-0.8	6.7	—
AD	E4	LN	5	99	umc1332	umc1221	3.1	-0.9	6.5	NutE, NHI
AD	E4	HN	7	119	bnlg339	umc1888	3.6	1.1	7.4	—

[a] Data for AD came from the experiment at E4 (Changping, CP, in 2008) as described by *Cai et al., 2011, Molecular Breeding*.
[b] A positive value means that Ye478 carried the allele for an increase in the trait, and a negative value.
[c] R^2 gives the percentage of the phenotypic variance explained by a putative locus.
[d] co-localized QTLs for AD and NUE- or RSA-related traits.

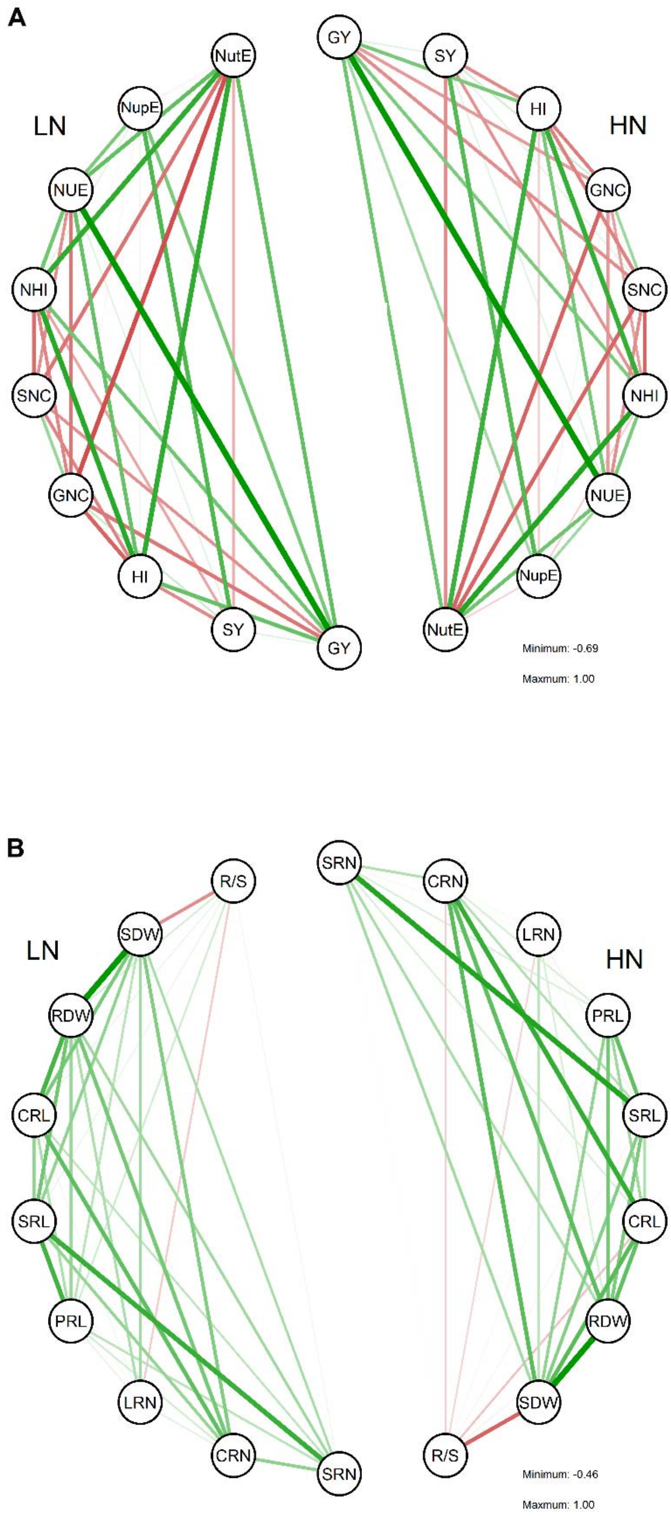

Figure S1. Network diagrams representing the phenotypic correlations between two traits within (A) NUE-related traits and (B) RSA-related traits based on their Pearson coefficients. Green and red lines represented the positive and negative correlation, respectively. Color scale and width of line represented the value of coefficients. HN and LN indicated the low-nitrogen and high-nitrogen levels, respectively.

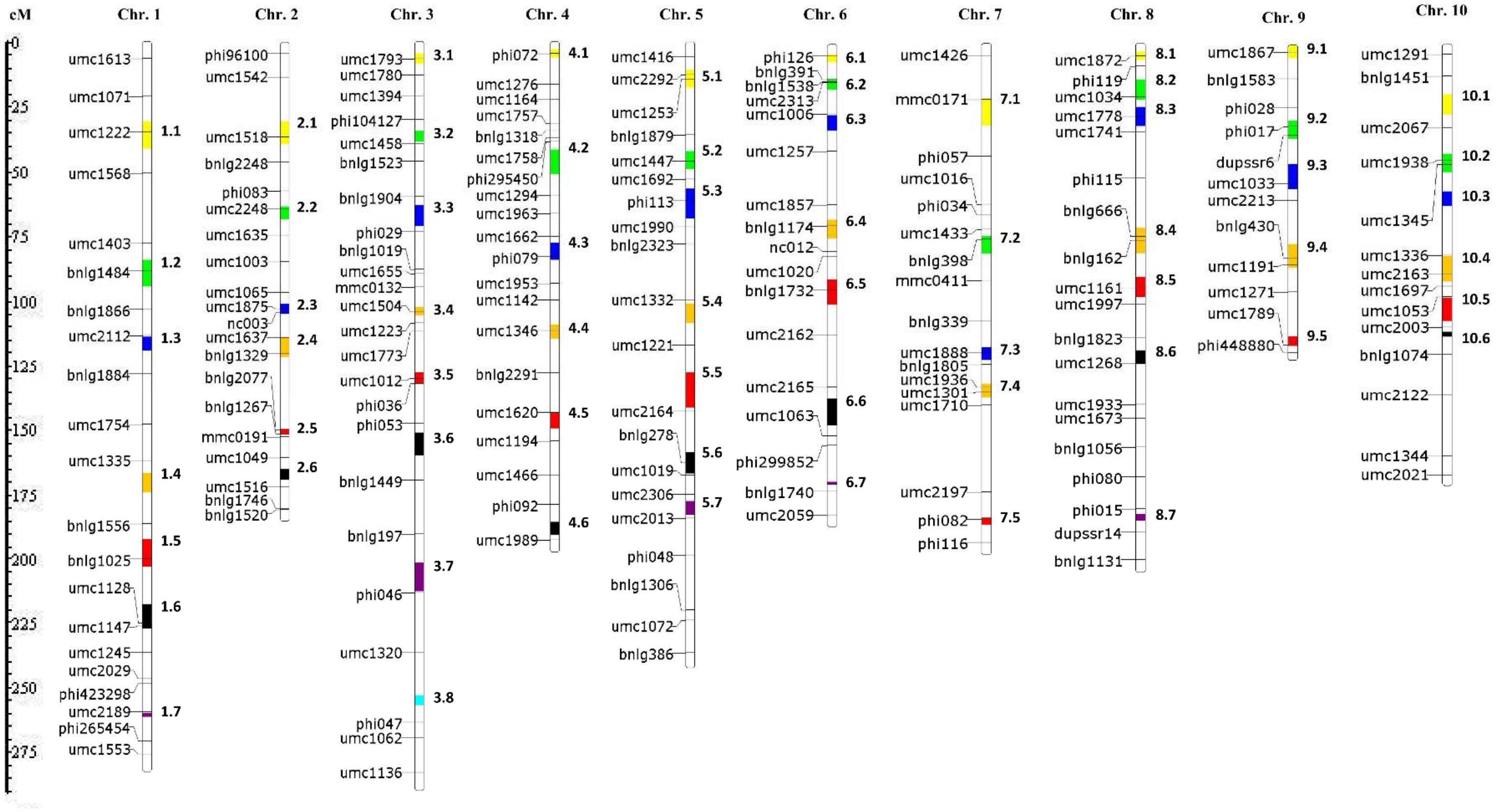

Figure S2. QTL clustering determined by MetaQTL software as described by Veyrieras et al. (2007). A vertical line represents the maker interval where the QTL was located. Each boxplot represents the identified QTL cluster, and the name is indicated on the right.

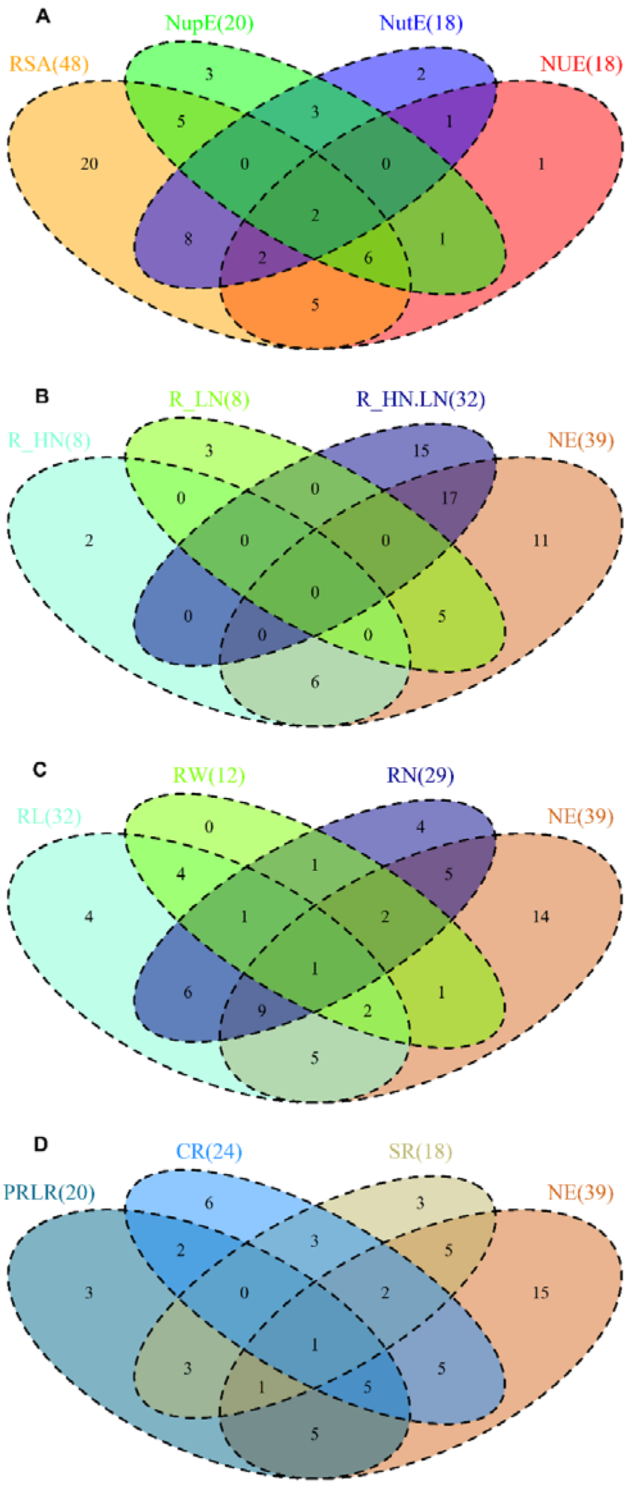

Figure S3. Numbers of QTL clusters for NUE- and RSA-related traits. (A) QTLs for eight root traits (CRN, CRL, SRN, SRL, PRL, LRN, RDW, R/S) were pooled as RSA-QTLs; (B) QTLs for NUE, NupE and NutE traits were pooled as NE-QTLs. RSA-QTLs were assigned into three groups according to their expression pattern (constitutive, HN-specific or LN-specific); (C) RSA-QTLs were assigned into three groups according to their trait types (root length, number and biomass). (D) RSA-QTLs were assigned into three groups according to root types (crown, seminal, primary and lateral roots). NE: traits of nitrogen efficiency including N use efficiency (NUE), N uptake efficiency (NupE) and N utilization efficiency (NutE). PRLR: combined traits for length of primary root (PRL) and lateral roots number (LRN).

References

Liu JC, Cai HG, Chu Q, Chen XH, Chen FJ, Yuan LX, Mi GH, Zhang FS. 2011. Genetic analysis of vertical root pulling resistance (VRPR) in maize using two genetic populations. *Molecular Breeding* **28**, 463-474.

Cai HG, Chu Q, Yuan LX, Liu JC, Chen XH, Chen FJ, Mi GH, Zhang FS. 2012. Identification of quantitative trait loci for leaf area and chlorophyll content in maize (*Zea mays* L.) under low nitrogen and low phosphorus supply. *Molecular Breeding* **30**, 251-266.
